# Regulatory Interplay between RNase III and Antisense RNAs in E. coli: the Case of AsflhD and FlhD, Component of the Master Regulator of Motility

**DOI:** 10.1128/mbio.00981-22

**Published:** 2022-08-24

**Authors:** Maxence Lejars, Joël Caillet, Eugenio Solchaga-Flores, Maude Guillier, Jacqueline Plumbridge, Eliane Hajnsdorf

**Affiliations:** a UMR8261, CNRS, Université Paris Cité, Institut de Biologie Physico-Chimique, Paris, France; National Institute of Child Health and Human Development (NICHD)

**Keywords:** asRNAs, RNase III, transcriptional attenuation, *flhD*, motility, *phoP*, *E. coli*

## Abstract

In order to respond to ever-changing environmental cues, bacteria display resilient regulatory mechanisms controlling gene expression. At the post-transcriptional level, this is achieved by a combination of RNA-binding proteins, such as ribonucleases (RNases), and regulatory RNAs, including antisense RNAs (asRNAs). Bound to their complementary mRNA, asRNAs are primary targets for the double-strand-specific endoribonuclease, RNase III. Taking advantage of our own and previously published transcriptomic data sets obtained in strains inactivated for RNase III, we selected several candidate asRNAs and confirmed the existence of RNase III-sensitive asRNAs for *crp*, *ompR*, *phoP*, and *flhD* genes, all encoding global regulators of gene expression in Escherichia coli. Using FlhD, a component of the master regulator of motility (FlhD_4_C_2_), as our model, we demonstrate that the asRNA AsflhD, transcribed from the coding sequence of *flhD*, is involved in the fine-tuning of *flhD* expression and thus participates in the control of motility.

## INTRODUCTION

Bacteria efficiently adapt to changes in their environment by sensing various signals and adjusting their genetic expression accordingly. Gene regulation occurs at all steps from DNA transcription to protein synthesis via a wide range of regulatory factors (both proteins and RNAs). *trans*-Encoded small RNAs (sRNAs) are regulators acting by imperfect base-pairing, often supported by RNA-binding protein (RBP) chaperones such as Hfq and ProQ (1). In contrast, antisense RNAs (asRNAs) are encoded in *cis* to their complementary target. Fewer asRNAs have been described compared to sRNAs, probably because of their high lability (as unprotected RNAs), their low conservation among species (e.g., asRNAs identified in a single study in Escherichia coli and Salmonella enterica revealed only 14% overlap [2]), and their low levels of expression (reviewed in references 3 and 4).

Initially, asRNAs were identified on mobile genetic elements (prophages and plasmids), in which their role is to control replication and partitioning. The importance of asRNAs was later demonstrated to extend to almost all kinds of biological processes (5), as in the case of type I toxin-antitoxin systems, in which the toxin mRNA is neutralized by an asRNA that induces degradation and/or inhibition of translation (6). Furthermore, the double-strand-specific RNase III has been known to be an important player in asRNA regulation, as in the case of the regulation of plasmid copy number and toxin-antitoxin systems (7, 8).

The mechanisms of action of asRNAs are diverse. They can negatively regulate transcription by interference due to the collision of two converging RNA polymerases (RNAPs) or by attenuation due, in some cases, to the stabilization of a terminator structure in the mRNA upon binding of the asRNA (9, 10). Moreover, despite their complementarity, the interaction of an asRNA with its target requires, in some cases, formation of an intermediate called “kissing complex” (7, 11). These interactions can have negative or positive consequences on gene expression since they induce modifications to the RNA secondary structure and/or physically interfere with the activity of other regulators (12, 13). In a surprisingly large number of cases, the mechanism by which a specific asRNA regulates its target remains unclear due, in part, to the impossibility of modifying the sequence of the asRNA independent of its target.

More recently, various genomic approaches have been used leading to the identification of hundreds to thousands of asRNAs and/or antisense transcription start sites (TSSs) in the transcriptome of E. coli. These approaches include genomic library overexpression (14), inhibition of Rho-dependent termination (15), mapping of transcriptional units (16), capture of double-stranded RNAs (17, 18) and enrichment of primary (19–21) or small transcripts (22). It is interesting to note that comparison of some of these data sets revealed only a modest overlap (19), enforcing the idea that asRNAs are difficult to identify and may not be well conserved even between related bacterial species.

Taking advantage of available transcriptomic data sets and of our previous study, during which a tailored transcriptome sequencing (RNA-seq) analysis was performed (23), we compared transcriptomes of an *rnc* mutant to its isogenic wt strain. We identified and validated the expression of four new asRNAs complementary to the coding sequence of genes *crp*, *ompR*, *phoP*, and *flhD*. We then investigated in detail how AsflhD controls the expression of *flhD*, one gene of the master regulator of swimming motility, FlhD_4_C_2_, and consequently affects the process of motility.

## RESULTS

### Characterization of asRNAs upon RNase III inactivation.

**FIG 1:**
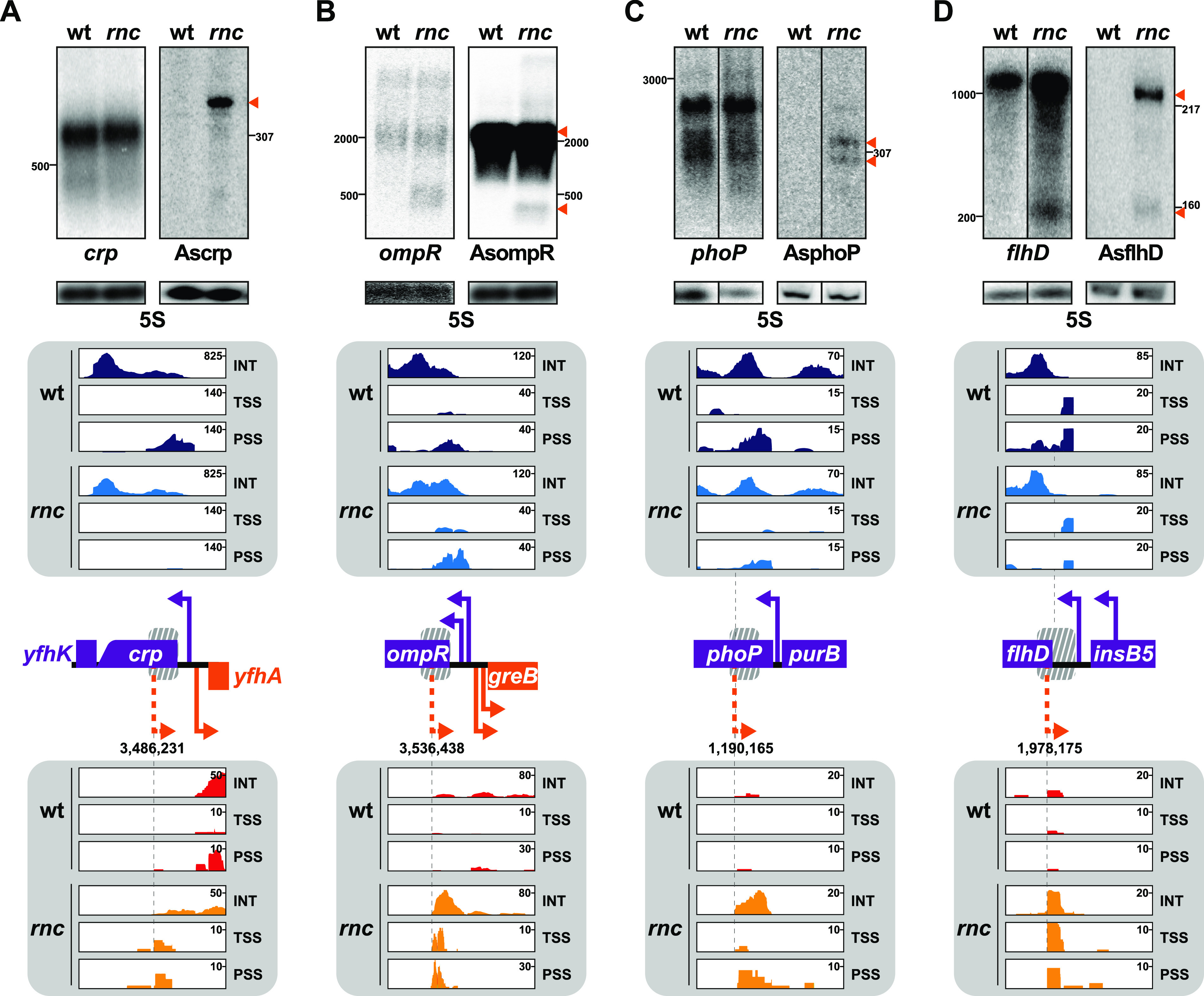
Antisense RNAs (asRNAs) accumulate upon RNase III inactivation. (A to D) Detection of asRNAs (dashed orange arrowheads) to *crp* (A), *ompR* (B), *phoP* (C), and *flhD* (D). RNAs extracted from wild-type (wt) strain N3433 and its *rnc* derivative IBPC633 were analyzed on agarose or denaturing acrylamide gels, and northern blots were probed by using pairs of complementary uniformly radiolabeled RNA probes to the same region of each target and a primer complementary to the 5S rRNA. To note, *crp* and AsompR were successively probed on the same membrane; thus, they share the same loading control. The membrane lanes shown for *phoP* and AsphoP (C) and AsflhD (D, left) correspond to the zero time points of the stability experiments presented in Fig. 2 (D and E) and Fig. S1A, respectively, which show the uncropped membranes. Transcriptome sequencing (RNA-seq) reads were aligned to the genome of reference (MG1655, GenBank identifier U00096.3) and visualized with Integrative Genomic Viewer (IGV) software, version 2.4.2. Antisense reads are oriented from left to right (5′ to 3′) independently of their genomic orientation. The fractions isolated during the RNA-seq analysis are color-coded: strands of the target gene in wt (dark blue) and in *rnc* mutant (light blue) and strands of the asRNA gene in wt (red) and in *rnc* mutant (orange). Reads corresponding to transcription start site (TSS), processing sites (PSS), and internal fragments (INT) are indicated. The scale for the absolute number of reads identified is indicated on the top right of each lane. Localization of open reading frames (ORFs) and known promoters (full bent arrows) and putative antisense promoters deduced from transcriptomic data sets (dashed bent orange arrows) are schematized on their respective loci, while the positions of the probes relative to the DNA sequence are indicated by a gray dashed box.

We previously performed a transcriptomic approach in E. coli K-12 (wt) and its *rnc*105 derivative strain (*rnc*) by tagging transcripts according to their 5′-phosphorylation status, allowing us to distinguish between 5′-triphosphate fragments (primary transcripts, TSS), 5′-monophosphate fragments (processed transcripts, PSS), and internal fragments resulting from the fragmentation (INT) (23). From this data set, we sorted the antisense reads to open reading frames (ORFs) that were enriched upon RNase III inactivation and checked whether they have been detected in independent data sets (as described in Text S1) (14, 16, 17, 19, 21). Four asRNAs complementary to gene coding for major transcriptional regulators were selected, i.e., asRNAs to *crp*, *ompR*, *phoP*, and *flhD.* Their coordinates are indicated in Table 1, and their expression was confirmed by northern blotting (Fig. 1). Of note, the identification of RNase III processing sites in the wt strain was usually not obvious since cleavage by RNase III presumably provoked the subsequent rapid degradation of the asRNA.

**TABLE 1:** Identification of asRNAs

Sense gene		asRNA			Detected in previous studies				
					asRNA reads			TSS only	
Name	Strand	Name	Genomic coordinates		(14)	(16)	(17)	(19)	(21)
			Starting from the TSS relative to E. coli genome U00096.3	Starting from the TSS relative to E. coli genome U00096.2					
*crp*	+	Ascrp	3,486,231 to 3,485,953	3,484,253 to 3,483,975	N	N	Y	N	N
*ompR*	−	AsompR	3,536,438 to 3,536,775	3,534,460 to 3,534,797	N	N	Y	Y^*a*^	Y^*a*^
*phoP*	−	AsphoP	1,190,165 to 1,190,508	1,189,388 to 1,189,731	N	N	Y^*a*^	Y^*a*^	Y^*a*^
*flhD*	−	AsflhD	1,978,175 to 1,978,395	1,976,199 to 1,976,419	Y^*a*^	N	Y	Y^*a*^	Y^*a*^

a Identical transcription start sites (TSSs) at ±1 nt.

10.1128/mbio.00981-22.1 TEXT S1 Supplemental Materials and Methods and uncropped northern blots. Download Text S1, DOCX file, 0.5 MB. Copyright © 2022 Lejars et al. 2022 Lejars et al. https://creativecommons.org/licenses/by/4.0/ This content is distributed under the terms of the Creative Commons Attribution 4.0 International license.

10.1128/mbio.00981-22.2 FIG S1 RNase III and AsflhD independently control the expression of *flhD*. (A to C) Total RNA was prepared from the wt strain (MG1655-B) (A), from the mutant decreasing the endogenous expression of AsflhD (P_AsflhD(−2)_; ML73) and their respective *rnc* derivatives (ML65 and ML75) (B), and from the mutant increasing endogenous expression of AsflhD (P_AsflhD(+1)_; ML241) (C) at different times after addition of rifampicin, and total RNA was subjected to northern blot analysis. The membranes were probed for *flhD* and M1 RNA. (A, top) The RNA probe used to detect *flhD* mRNA (5′-probe) is represented in black within the *flhD* locus (*flhD* in purple and AsflhD promoter in orange). Full-length *flhD* mRNA (*flhD*_FL_) and short *flhD* 5′-UTR RNA (*flhD*_p_) are indicated. The decay rate of *flhD*_FL_ mRNA was calculated as described in the Material and Methods section. Download FIG S1, EPS file, 2.7 MB. Copyright © 2022 Lejars et al. 2022 Lejars et al. https://creativecommons.org/licenses/by/4.0/ This content is distributed under the terms of the Creative Commons Attribution 4.0 International license.

The *crp* gene encodes the major regulator of carbon catabolite repression, and it was shown previously to be transcriptionally regulated by the recruitment of the RNAP to a divergent and overlapping TSS located 3 nt upstream from the *crp* TSS (24, 25) encoding the divergently expressed gene *yfhA*. We observed antisense reads validating this previously characterized TSS in the wt strain. In addition, antisense reads complementary to the ORF and 5′-untranslated region (5′-UTR) of the *crp* mRNA accumulate in the *rnc* mutant (Fig. 1A; Table 1), and we predicted an additional TSS to be located 112 nt downstream from the start codon of *crp*. Northern blot analysis revealed that this asRNA, here referred to as Ascrp, is stabilized in the *rnc* mutant as a fragment longer than 307 nt.

The *ompR* gene encodes the response regulator of a two-component system involved in cell wall homeostasis and response to low pH, EnvZ-OmpR (26–29). From our and previous data sets, we predicted a TSS located 147 nt downstream from the start codon of *ompR* (Fig. 1B; Table 1). Northern blotting with complementary probes corresponding to the 5′-end of the *ompR* ORF confirmed the presence of the *ompR-envZ* dicistronic mRNA and revealed a long asRNA, here referred to as AsompR, of about 2,000 nt, which is likely to also encode the divergently expressed *greB* gene. In addition, smaller fragments (less than 500 nt) are detected in the *rnc* mutant for both *ompR* and AsompR (Fig. 1B), which could correspond to a stable duplex between *ompR* and AsompR cleaved by RNase III in the wt strain.

The *phoP* gene encodes the response regulator of the PhoQ-PhoP two-component system, involved in response to low magnesium and in cell wall homeostasis (30, 31). From our and previous data sets, we predicted a TSS to be located 282 nt downstream from the *phoP* start codon (Fig. 1C; Table 1). Northern blots hybridized with complementary probes corresponding to the 5′-end of the *phoP* ORF confirmed the accumulation of two fragments (AsphoP) about 300 to 320 nt long in the *rnc* mutant (Fig. 1C).

The *flhDC* operon encodes the master regulator of motility, FlhD_4_C_2_ (32). Our and previous data sets allowed us to predict a TSS to be located 22 nt downstream from the *flhD* start codon (Fig. 1D; Table 1). Northern blot analysis with probes specific to the 5′-UTR of *flhD* confirmed the accumulation of an asRNA to *flhD* in the *rnc* mutant, here referred to as AsflhD, as a major fragment of about 220 nt and a minor fragment of about 160 nt upon RNase III inactivation. At the same time, an increase in the amount of the full-length *flhD* mRNA and the stabilization of a *flhD* fragment with an approximate size of 220 nt were observed in the *rnc* mutant (Fig. 1D), which could correspond to a double-stranded RNA formed between *flhD* and AsflhD RNAs.

The four identified asRNAs fragments are likely to be processed by RNase III since they are only visible in the *rnc* strain. However, we cannot exclude the possibility that RNase III is involved indirectly at another stage in the regulation of their transcription. Crp, OmpR, PhoP, and FlhD are major regulators of gene expression involved in the control of large regulons (RegulonDB version 10.5 [33]), which are conserved among gammaproteobacteria (34). We wondered whether these asRNAs and RNase III have a functional regulatory role on their targets and thus affect cell physiology. We investigated asRNAs to *phoP* and *flhD*, two regulators whose expression is known to be tightly controlled at both the transcriptional and post-transcriptional levels (35, 36).

### Regulation of *phoP* and AsphoP by RNase III.

**FIG 2:**
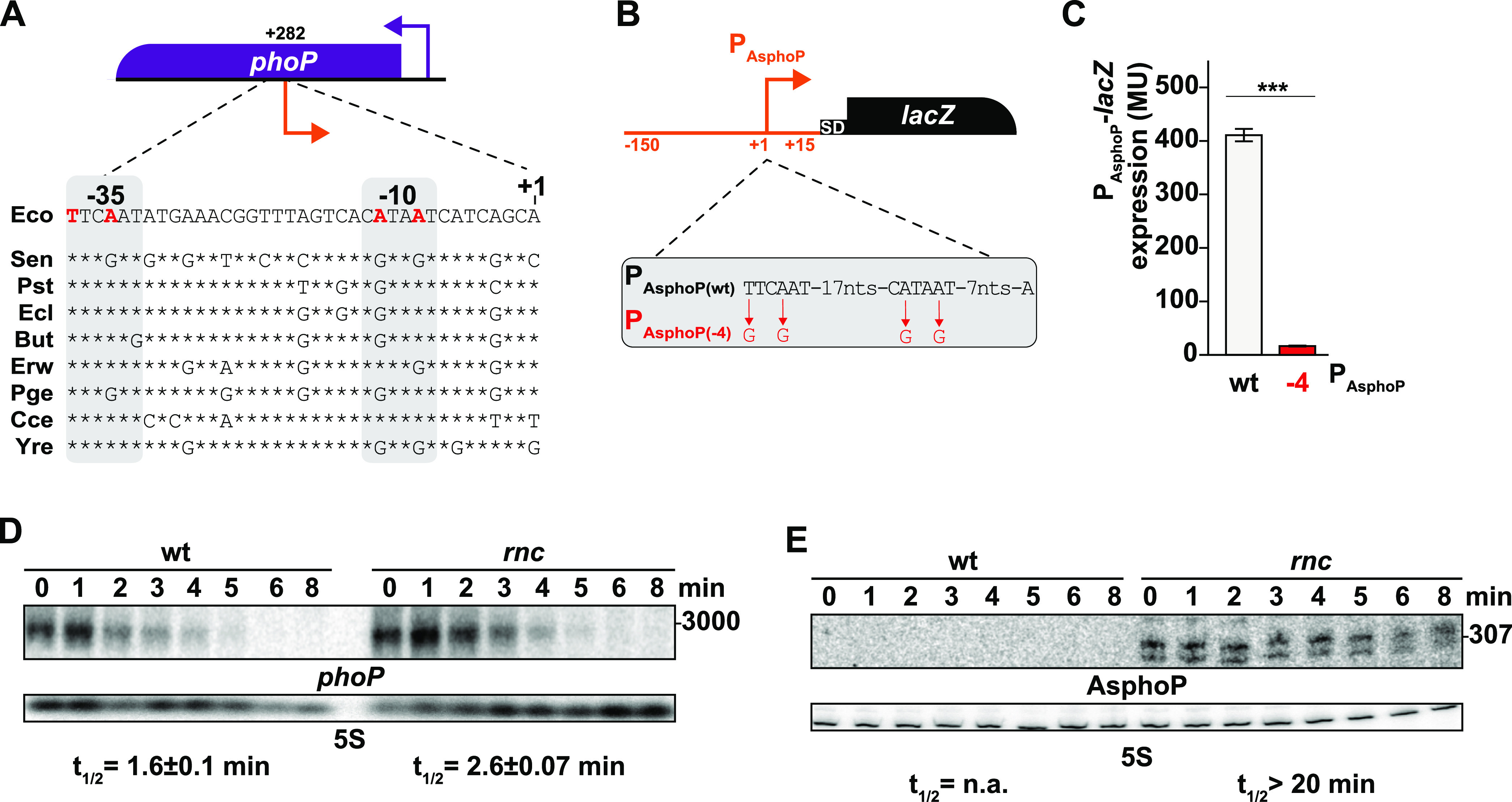
Regulation of AsphoP and *phoP* levels by RNase III. (A) Genetic structure of the *phoP* locus and alignment of the promoter sequence of AsphoP from selected bacterial species. P_AsphoP_ (orange bent arrow) is indicated relative to the translation start of *phoP* mRNA (+282). Sequences correspond to the following genomes: Eco, Escherichia coli MG1655 (NC_000913.3); Sen, Salmonella enterica LT2 (CP014051.2); Pst, Pantoea stewartia ZJ-FGZX1 (CP049115.1); Ecl, Enterobacter cloacae NH77 (CP040827.1); But, Buttiauxella sp. 3AFRM03 (CP033076.1); Erw, *Erwinia* sp. J780 (CP046509.1); Pge, Pluralibacter gergoviae (LR699009.1); Cce, Clostridium cellulovorans 743B (CP002160.1); and Yre, Yokenella regensburgei W13 (CP050811.1). Nucleotides mutated to inactivate P_AsphoP_ are shown in red, stars represent conserved nt relative to Eco, and the −35 and −10 motifs of AsphoP are highlighted in gray. (B) Genetic structure of the transcriptional P_AsphoP_-*lacZ* reporter fusion (P_AsphoP(wt)_ in MG2118). Mutations repressing activity of the AsphoP promoter (P_AsphoP(−4)_ in MG2120) are in red. (C) Expression of β-galactosidase activity (given as Miller units [67]) was determined from the P_AsphoP(wt)_-*lacZ* and P_AsphoP(-4)_*-lacZ* fusions in the wt strain. The values are the means of three biological replicates for each strain, and the bars indicate the standard deviations. Statistical significance was determined by analysis of variance (ANOVA). ***, *P* ≤ 0.001. (D and E) Total RNA was prepared from samples taken from the wt strain (N3433) and its *rnc* derivative (IBPC633) at different times after addition of rifampicin and was subjected to northern blot analysis. The membranes were probed successively for *phoP* or AsphoP and 5S. The decay rate of *phoP* mRNA was calculated as described in the Materials and Methods section.

Candidate consensus −10 and −35 sequences were predicted upstream of AsphoP TSS (Fig. 2A). To validate this promoter, we constructed a P_AsphoP_-*lacZ* transcriptional fusion containing 150 nt before and 15 nt after the putative TSS of AsphoP, with the wt sequence (P_AsphoP(wt)_) or mutations (P_AsphoP(−4)_) decreasing the agreement with the consensus in the predicted −35 and −10 boxes (Fig. 2B). The mutated AsphoP promoter (P_AsphoP(−4)_) strongly decreased the expression of P_AsphoP_-*lacZ* (20-fold), confirming it as an endogenous AsphoP promoter (Fig. 2C). Furthermore, inactivation of RNase III led to an increase (1.6-fold) of *phoP* mRNA stability (Fig. 2D) and revealed that AsphoP is much more stable than *phoP* mRNA in the *rnc* mutant (Fig. 2E). In summary, we confirmed that AsphoP is transcribed from the predicted promoter and that RNase III negatively affects the expression of *phoP* and AsphoP.

Sequence comparison with other bacterial species showed that although the region of the AsphoP promoter is moderately well conserved, there are several A to G substitutions in the −10 box at positions −9 and −12, suggesting that this promoter may be inactive in the compared genomes (Fig. 2A). This, in turn, implies that, if AsphoP has any function, it could be limited to E. coli K-12 and has been counterselected in these other species or, more likely, represents a novel, evolving trait.

### Physiological expression of AsflhD.

**FIG 3:**
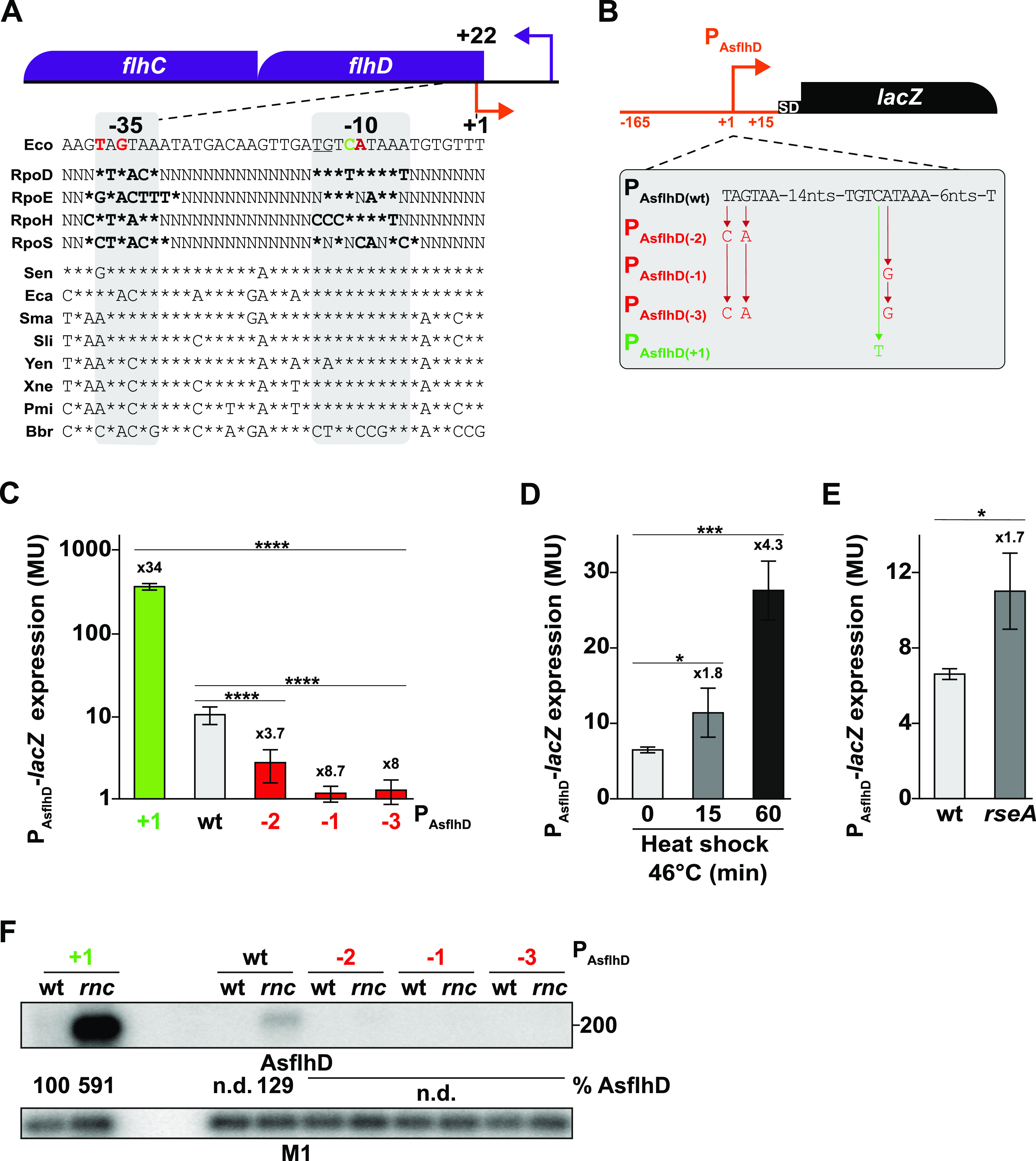
Transcriptional regulation of AsflhD. (A) Genetic structure of the *flhD* locus and alignment of the promoter sequence of AsflhD with the consensus sequences for the RpoD-, RpoE-, RpoH-, and RpoS-dependent promoters (38, 39, 74) and with eight Eubacterial species showing 49 to 92% FlhD identity with E. coli (51). The position of the promoter of AsflhD (orange bent arrow) is indicated relative to the *flhD* translation start of *flhD* (+22). Sequences correspond to the following bacteria: Eco, Escherichia coli MG1655 (NC_000913.3); Sen, Salmonella enterica Typhimurium (D43640); Eca, Erwinia carotovora (AF130387); Sma, Serratia marcescens (AF077334); Sli, Serratia liquefaciens (Q7M0S9); Yen, Yersinia enterocolitica (AF081587); Xne, Xenorhabdus nematophilus (AJ012828); Pmi, Proteus mirabilis (U96964); and Bbr, Bordetella bronchiseptica (U17998). Bases identical to the E. coli sequence are shown with asterisks. Gray highlighting indicates the bases corresponding to the consensus for a TG-extended *rpoD* promoter (37). (B) Genetic structure of the transcriptional P_AsflhD_-*lacZ* reporter fusion and location of mutations altering the AsflhD promoter. (C) Expression of β-galactosidase from P_AsflhD_-*lacZ* fusions, wt (P_AsflhD(wt)_, MG2114-P_AsflhD_), and derivatives carrying the P_AsflhD_ mutations (P_AsflhD(−2)_, ML239; P_AsflhD(−1)_, ML604; P_AsflhD(−3)_, ML605; and P_AsflhD(+1)_, ML218) at 37°C. Of note, P_AsflhD(+1)_ leads to the replacement of the 12th codon GAC (aspartic acid) to the codon AAC (asparagine), P_AsflhD(−2)_ leads to the replacement of the 19th codon CUA to the synonymous codon UUG, and P_AsflhD(−1)_ leads to the replacement of the 11th codon UAU to the synonymous codon UAC, while P_AsflhD(−3)_ combines the effect of the two previous mutations. (D) Expression of β-galactosidase in P_AsflhD(wt)_-*lacZ* before (30°C, *t* = 0) and after 15 and 60 min of upshift (46°C). (E) Expression of β-galactosidase in P_AsflhD(wt)_-*lacZ* in the *rseA* mutant derivative (ML279) at 37°C. The values are means of three biological replicates for each strain, and the bars indiate the standard deviations. Statistical significance was determined by ANOVA. *, *P* ≤ 0.05; ***, *P* ≤ 0.001; ****, *P* ≤ 0.0001. (F) Mutations in red were introduced in the endogenous *flhD*-AsflhD locus to reduce the activity of the AsflhD promoter (P_AsflhD(−2)_, ML73; P_AsflhD(−1)_, ML609; and P_AsflhD(−3)_, ML610) and to increase its activity in green (P_AsflhD(+1)_, ML241). Strains wt (MG1655-B), P_AsflhD(−2)_ (ML73), P_AsflhD(−1)_ (ML609), P_AsflhD(−3)_ (ML610), P_AsflhD(+1)_ (ML241), and their *rnc* derivatives (ML65, ML75, M613, ML614, and ML341, respectively) were grown at 37°C until mid-log phase. Total RNA was analyzed by northern blotting. The membrane was probed successively for AsflhD and for M1 RNA (377 nt, highly stable catalytic component of the RNase P, used as a loading control [75]). n.d., not determined.

Candidate −10 and −35 boxes were identified upstream from the putative TSS of AsflhD and sequence alignment of this region in other enterobacteria shows a good conservation of a promoter with an extended −10 5′-TG box (37), suggesting that AsflhD expression is conserved among enterobacteria (Fig. 3A). To validate the predicted promoter, a P_AsflhD_-*lacZ* transcriptional fusion (P_AsflhD(wt)_) was constructed containing 165 nt before and 15 nt after the putative TSS of AsflhD (Fig. 3B). This fusion showed a relatively low level of β-galactosidase activity (10MU; Fig. 3C). Its expression was strongly increased (34-fold) when the −10 motif was improved toward the RpoD consensus (P_AsflhD(+1)_), while mutating the −35 and −10 to less consensus sequences (P_AsflhD(−2)_, P_AsflhD(−1)_, and P_AsflhD(−3)_) decreased expression (3.7- to 8.7-fold), hence validating the predicted promoter (Fig. 3C). It should be noted that mutations were designed to be used in the endogenous *flhD* locus and chosen to minimally affect the coding sequence of *flhD* and to avoid introduction of rare codons. However, the P_AsflhD(+1)_ mutation produces an aspartate to asparagine change at position 12 of FlhD (D12N), which may affect FlhD function (see below). The low level of expression made us wonder whether AsflhD may be expressed using an alternative sigma factor. Heat shock increased P_AsflhD_-*lacZ* expression 1.8-fold after 15 min and 4.3-fold after 60 min (Fig. 3D). Comparison of P_AsflhD_ with consensus sequences for the two heat-shock sigma factors, σ^H^ (RpoH) and σ^E^ (RpoE) (38, 39), shows better correlation with the σ^E^ consensus than with σ^H^ (Fig. 3A), suggesting that RpoE could be involved in the transcription from the P_AsflhD_.

Hence, we examined whether P_AsflhD_ is under the control of RpoE by using a strain deleted for *rseA* (anti-sigma factor inhibitor of RpoE), which leads to the strong induction of the RpoE regulon (40, 41). Deletion of *rseA* increased the expression of the wt P_AsflhD_-*lacZ* fusion (Fig. 3E, 1.7-fold), comparable with the effect of the heat shock at 46°C, known to induce the RpoE regulon (42). Thus, our results suggest that AsflhD is more expressed at high temperature and when the RpoE sigma factor is activated.

To further characterize AsflhD, the P_AsflhD(−2)_, P_AsflhD(−1)_, P_AsflhD(−3)_, and P_AsflhD(+1)_ mutations were introduced at the endogenous *flhD* locus, and the expression of AsflhD was examined by northern blotting (Fig. 3F). No AsflhD was detected in the *rnc* derivatives of strains with the three mutations reducing the expression of AsflhD. Conversely, AsflhD overexpression from the P_AsflhD(+1)_ mutation led to the detection of a faint smear in the wt strain and to the much greater accumulation of AsflhD in the *rnc* mutant (4.6-fold relative to the *rnc* mutant containing the P_AsflhD(wt)_). In summary, we have identified the promoter of AsflhD and shown that mutations in the promoter of AsflhD can be used as tools to study the function of AsflhD at the *flhD* locus.

### Processing of AsflhD.

**FIG 4:**
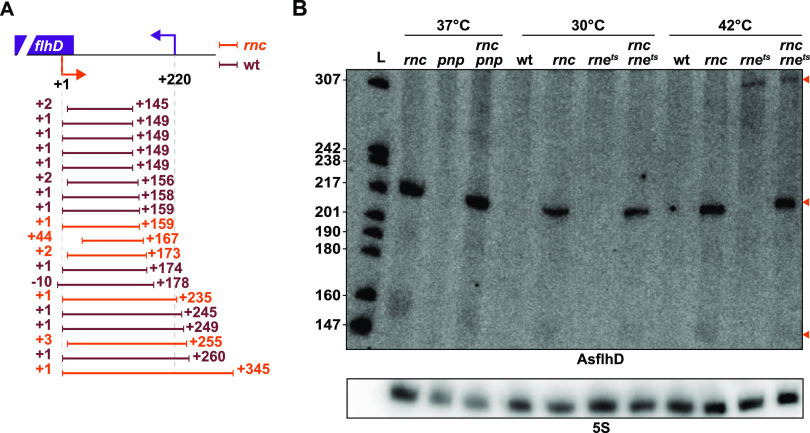
Characterization of AsflhD. (A) cRT-PCR fragments were cloned and individually sequenced. Each line represents a single transcript identified from the wt strain N3433 (dark red) or from the *rnc* mutant IBPC633 (orange). The 5′- and 3′-end positions are indicated relative to the TSS of AsflhD. (B) The wt strain (N3433) and its derivatives, *pnp*, *rnc*, *pnp*-*rnc*, *rne^ts^*, and *rnc*-*rne^ts^* mutants (N3433-*pnp*, IBPC633, IBPC633-*pnp*, N3431, and IBPC637, respectively), were grown at 37°C until mid-log phase. Where indicated, the cells were grown at 30°C and submitted to a heat shock at 42°C for 15 min in order to inactivate RNase E in the strain carrying the thermosensitive *rne*^ts^ allele. Total RNA was analyzed by northern blotting. The membrane was probed successively for AsflhD and 5S. AsflhD fragments are indicated by orange arrowheads.

Circular RT-PCR (cRT-PCR) experiments confirmed that AsflhD is expressed in both the wt and *rnc* strains from P_AsflhD_ with heterogeneous AsflhD 3′-ends extending up to 345 nt in the *rnc* mutant (Fig. 4A). Surprisingly, no 220-nt-long RNA was detected in the mutant by cRT-PCR, while a 149-nt-long fragment was found several times exclusively in the wt strain, which might be an intermediate in the degradation of AsflhD, *e.g.*, via RNase E (see below). Surprisingly, we did not detect 220-nt RNAs observed by northern blotting in the *rnc* strain. As this species is likely to be present as double-stranded RNA with the processed *flhD* mRNA (as described below), we suspect that it is less efficiently ligated during the initial step of the cRT-PCR, as previously reported (see Materials and Methods) (43). We investigated the degradation of AsflhD by other RNases. Inactivation of the major endonuclease RNase E allowed the detection of a 300-nt fragment (independently of the presence of RNase III), whereas the loss of the exoribonuclease polynucleotide phosphorylase (PNPase) had no effect on AsflhD degradation (Fig. 4B). Hence, RNase III and RNase E are independently involved in the rapid turnover of AsflhD.

**FIG 5:**
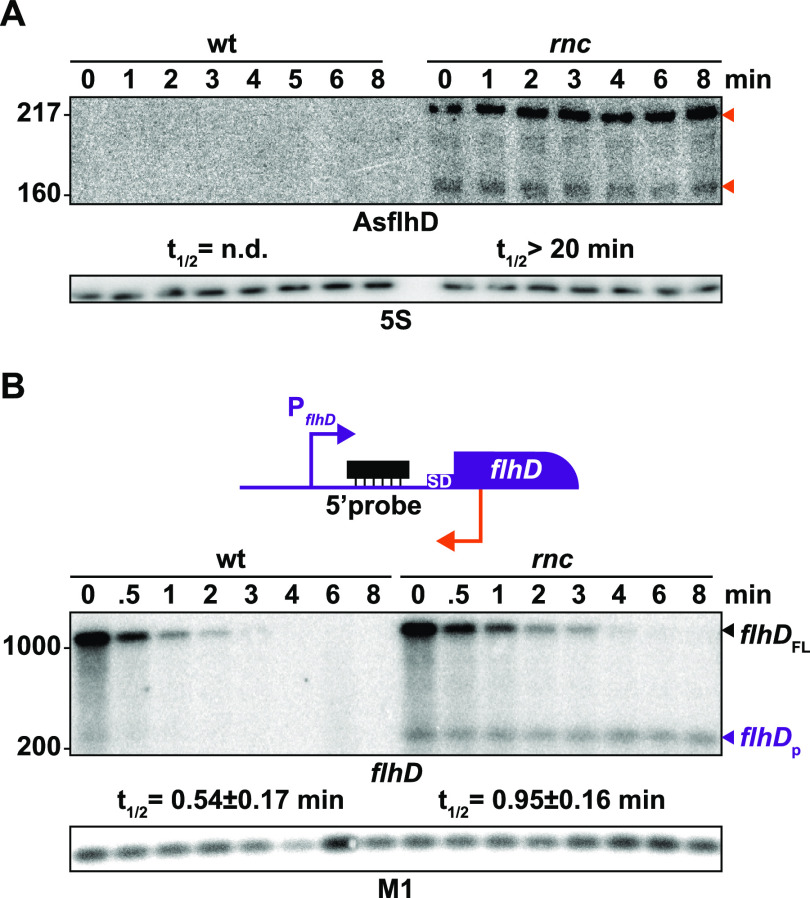
Repression of AsflhD and flhD expression by RNase III *in vivo*. (A) Total RNA was prepared from the wt strain MG1655-B and its *rnc* derivative ML65 at different times after addition of rifampicin, and total RNA was subjected to northern blot analysis. Membranes were probed for AsflhD and 5S (A) or *flhD* and M1 RNA (B). AsflhD fragments are indicated by orange arrowheads. (B, top) The RNA probe used to detect *flhD* mRNA (5′-probe) is represented in black within the *flhD* locus (*flhD* in purple and AsflhD promoter in orange). (B, bottom) Transcripts corresponding to full-length *flhD* mRNA (*flhD*_FL_) and short *flhD* 5′-UTR RNA (*flhD*_p_) are indicated by arrowheads. The decay rate of *flhD*_FL_ mRNA was calculated as described in the Materials and Methods section.

**FIG 6:**
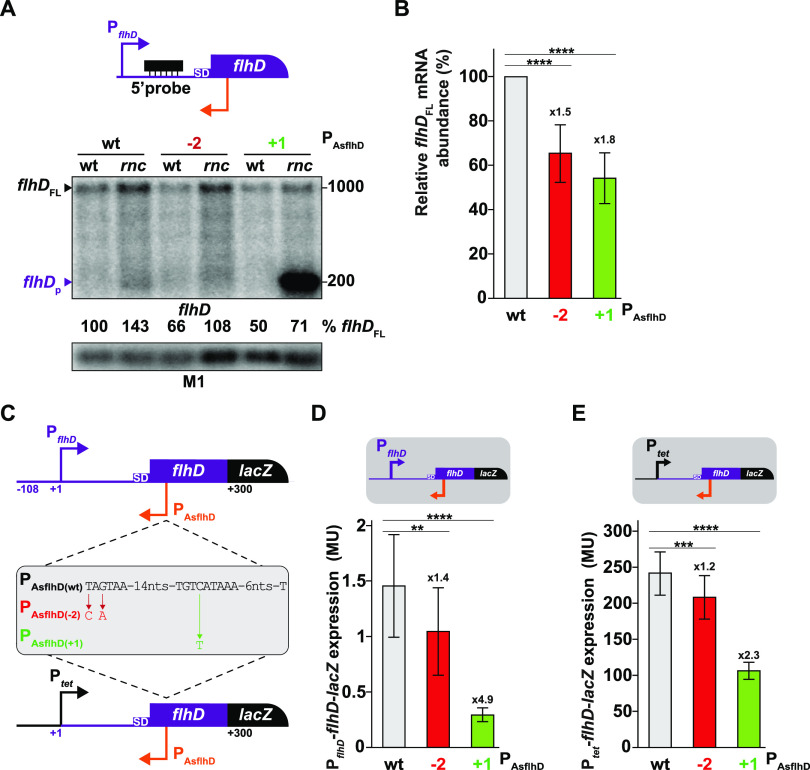
AsflhD regulates the expression of *flhD*. (A, top) The RNA probe used to detect *flhD* mRNA (5′-probe) is represented in black relative to the *flhD* locus (*flhD* in purple and AsflhD promoter in orange). (A, bottom) Total RNA was extracted from the wt (MG1655-B) and endogenous AsflhD promoter mutants (P_AsflhD(−2)_ in ML73 and P_AsflhD(+1)_ in ML241) and their *rnc* mutant derivatives (ML65, ML75, and ML341, respectively) and subjected to northern blot analysis. The membrane was probed successively for *flhD* and for M1 RNA. (B) Average *flhD* mRNA abundance in the P_AsflhD(−2)_ (red) and P_AsflhD(+1)_ (green) mutants relative to the wt strain (gray), as shown in panel A, was calculated as the mean of five biological replicates. (C) Genetic structures of the P*_flhD_*-*flhD*-*lacZ* (ML219) and P*_tet_*-*flhD*-*lacZ* reporter fusions (ML233) and their derivatives containing the mutations leading to either decreased expression (P_AsflhD(−2)_, ML221 and ML235, respectively, in red) or increased expression (P_AsflhD(+1)_, ML226 and ML237, respectively, in green) of AsflhD. (D, E) Expression of P*_flhD_*-*flhD*-*lacZ* (D) and P*_tet_*-*flhD*-*lacZ* (E) reporter fusions (gray) and their derivatives are given as β-galactosidase activity. The values are means of 10 biological replicates for each strain, and the bars indicate standard deviations. Statistical significance was determined by ANOVA. **, *P* ≤ 0.01; ***, *P* ≤ 0.001; ****, *P* ≤ 0.0001.

To understand the role of RNase III in the degradation of *flhD* and AsflhD RNAs, we analyzed the effect of RNase III inactivation on the stability of both RNAs. In the *rnc* mutant, the major 220-nt-long AsflhD transcript and minor 160-nt-long transcript were highly stable, while both the amount and the stability of the full-length *flhDC* mRNA (here referred to as *flhD*_FL_) increased 2-fold (Fig. 5A and B). In addition, the 5′-UTR probe used (Fig. 1D and 5B, top; Table S1) detected a 220-nt-long 5′-UTR *flhD* RNA fragment (here referred to as *flhD*_p_), complementary to AsflhD, which is also highly stable in the *rnc* strain. The interaction of *flhD*_p_ with AsflhD is expected to generate a double-stranded RNA (Fig. 4A), the degradation of which depends on RNase III. Supporting this hypothesis, we could not detect *flhD*_p_ RNA in a strain with RNase III inactivated and in which the endogenous AsflhD expression was reduced (P_AsflhD(−2)_), while *flhD*_p_ RNA accumulates when AsflhD expression was increased (P_AsflhD(+1)_; Fig. 6A; Fig. S1B). We further confirmed the interaction and cleavage by RNase III of AsflhD and *flhD in vitro*. A 308-nt-long *flhD* transcript corresponding to the 5′-UTR and part of the ORF of the *flhD* mRNA and a 256-nt-long AsflhD asRNA were synthesized and labeled at their 5′-ends. These two RNAs form a duplex when present in equimolar concentrations (Fig. S2A), which is completely degraded upon addition of RNase III (Fig. S2B). Remarkably, under the same condition, RNase III cleaves the individual RNAs independently at two sites on AsflhD and at four major sites on *flhD* (Fig. S2C and D). These cleavage sites are located within regions able to form a secondary structure on each molecule (44, 45) (Fig. S2C and D). RNase III is thus able to cleave both AsflhD and *flhD* RNAs *in vitro*, at specific sites but is also able to drive the complete degradation of the *flhD*/AsflhD duplex. These results support a dual role of RNase III in the processing of *flhD* mRNA via the binding of AsflhD or independently of AsflhD.

10.1128/mbio.00981-22.3 FIG S2 *In vitro* cleavage of AsflhD and *flhD* RNAs by RNase III. 5′-Radiolabeled AsflhD RNA (308 nt) was incubated with increasing concentrations of *flhD* mRNA (256 nt) under conditions referred to as “native” and “full” RNA duplex conditions (see Materials and Methods). (A, B) Native AsflhD-*flhD* complexes were formed at 37°C for 5 min in TMN buffer (20 mM Tris acetate, pH 7.5, 10 mM magnesium acetate, 100 mM sodium acetate), and full duplexes were obtained after a denaturation-annealing treatment in TE buffer (2 min 90°C and 30 min 37°C) before loading on native polyacrylamide gels to control for hybridization efficiency (A) or *in vitro* processing by RNase III (B). (B) RNase III digestion of complexed AsflhD and *flhD* in native conditions was performed at 37°C in TMN buffer containing 1μg tRNA with increasing concentration of RNase III per sample. (C, D) Uncomplexed AsflhD and *flhD* RNAs were digested under the same conditions with I unit RNAse III. Samples were analyzed on 8% polyacrylamide-urea gels. 5′-Radiolabeled *flhD* (C) and AsflhD (D) were cleaved by RNase III *in vitro* at four and two main sites, respectively (represented by a numbered black arrow). Mapping of the main *in vitro* cleavage sites of RNase III on *flhD* and AsflhD is indicated on their predicted secondary structures (according to reference 45 and Vienna RNA websuite [44]) with the position of the main RNase III cleavage sites indicated relative to the TSS. The localization of RNase III cleavage sites (black arrows) was performed by comparing the cleavage fragment size relative to an alkaline RNA ladder (NaOH) obtained by partial hydrolysis in NaOH of the same labeled RNAs and radioactive markers. Download FIG S2, EPS file, 2.6 MB. Copyright © 2022 Lejars et al. 2022 Lejars et al. https://creativecommons.org/licenses/by/4.0/ This content is distributed under the terms of the Creative Commons Attribution 4.0 International license.

10.1128/mbio.00981-22.10 TABLE S1 Strains, plasmids, and primers used in this study. Download Table S1, DOCX file, 0.04 MB. Copyright © 2022 Lejars et al. 2022 Lejars et al. https://creativecommons.org/licenses/by/4.0/ This content is distributed under the terms of the Creative Commons Attribution 4.0 International license.

### Regulation of *flhD* expression by AsflhD.

We next determined the effect of increased or decreased expression of AsflhD, in *cis*, on *flhD* expression by following *flhD* mRNA abundance and stability in strains carrying the endogenous P_AsflhD_ mutations described above. While a decrease in *flhD* mRNA abundance results from both decreased AsflhD (1.5-fold in P_AsflhD(−2)_, 1.6-fold in P_AsflhD(−1)_, and 1.2-fold in P_AsflhD(−3)_) and increased AsflhD expression (1.8-fold in P_AsflhD(+1)_; Fig. 6A and B; Fig. S3A), the stability of *flhD* mRNA was not significantly affected in either the P_AsflhD(−2)_ or P_AsflhD(+1)_ mutant (Fig. S1A to C).

10.1128/mbio.00981-22.4 FIG S3 Decreasing or increasing the expression of AsflhD reduces *flhD* expression. (A) Total RNA samples from strains wt (MG1655-B) and from endogenous AsflhD mutant promoters (P_AsflhD(−2)_, ML73; P_AsflhD(+1)_, ML241; P_AsflhD(−1)_, ML609; and P_AsflhD(−3)_, ML610) were subjected to northern blot analysis. The membrane was probed for *flhD* and M1 RNA; lanes were taken from the same membrane and uncropped membranes are shown in the Text S1. (A, top) The RNA probe used to detect *flhD* mRNA (5′-probe) is represented in black within the *flhD* locus (*flhD* in purple and AsflhD promoter in orange). Full-length *flhD* mRNA (*flhD*_FL_) is indicated. (B, C) Expression of (B) P*_flhD_*-*flhD*-*lacZ* (ML219) and (C) P*_tet_*-*flhD*-*lacZ* (ML233) reporter fusions (gray) and their derivatives carrying the mutations leading to either decreased expression (P_AsflhD(−2)_, ML221 and ML235, respectively, in red) or increased expression (P_AsflhD(+1)_, ML226 and ML237, respectively, in green) of AsflhD were analyzed at *A*_600_ ≈ 1. The values are the means of seven biological replicates for each strain, and the bars indicate standard deviations. Statistical significance was determined by ANOVA. **, *P* ≤ 0.01; ***, *P* ≤ 0.001; ****, *P* ≤ 0.0001. Download FIG S3, EPS file, 2.0 MB. Copyright © 2022 Lejars et al. 2022 Lejars et al. https://creativecommons.org/licenses/by/4.0/ This content is distributed under the terms of the Creative Commons Attribution 4.0 International license.

A transcriptional/translational (P*_flhD_*-*flhD*-*lacZ*) and a translational *flhD-lacZ* reporter fusion under the control of a constitutive promoter (P*_tet_*-*flhD*-*lacZ*) encompassing the 5′-UTR and the first 34 amino acids of FlhD (which includes P_AsflhD_) were introduced at the *lacZ* chromosomal locus (Fig. 6C). Mutations in P_AsflhD_ resulting in decreased (P_AsflhD(−2)_) and increased expression (P_AsflhD(+1)_) of AsflhD were also introduced into both fusions. Both mutations reduced expression of both fusions, but the effect of the AsflhD-overexpressing mutation (P_AsflhD(+1)_) was greater (4.9-fold decrease of the fusion driven by the wt *flhD* promoter and 2.3-fold on the P*_tet_-driven* version) than the effect of the promoter-down mutation (1.4-to 1.2-fold in P_AsflhD(−2)_; Fig. 6D and E). These results, obtained during the exponential phase of growth, were confirmed by measurements in late exponential phase (*A*_600_ ≈ 1; Fig. S3B and C) when *flhD* expression, from the P*_flhD_* promoter, increases (as previously reported [46]). It can also be noted that AsflhD overexpression has a greater impact on *flhD-lacZ* expression from the native *flhD* promoter (Fig. 6D) than on *flhD* mRNA abundance (Fig. 6A) or *flhD-lacZ* expression from the P*_tet_* promoter (Fig. 6E).

In brief, reducing or increasing the expression of AsflhD reduces *flhD* expression at the translational and mRNA levels, while not appreciably affecting the stability of the *flhD* mRNA. Hence, this suggests that a native intermediate level of AsflhD expression is required for optimal *flhD* expression.

### AsflhD represses *flhD* expression in *trans*.

**FIG 7:**
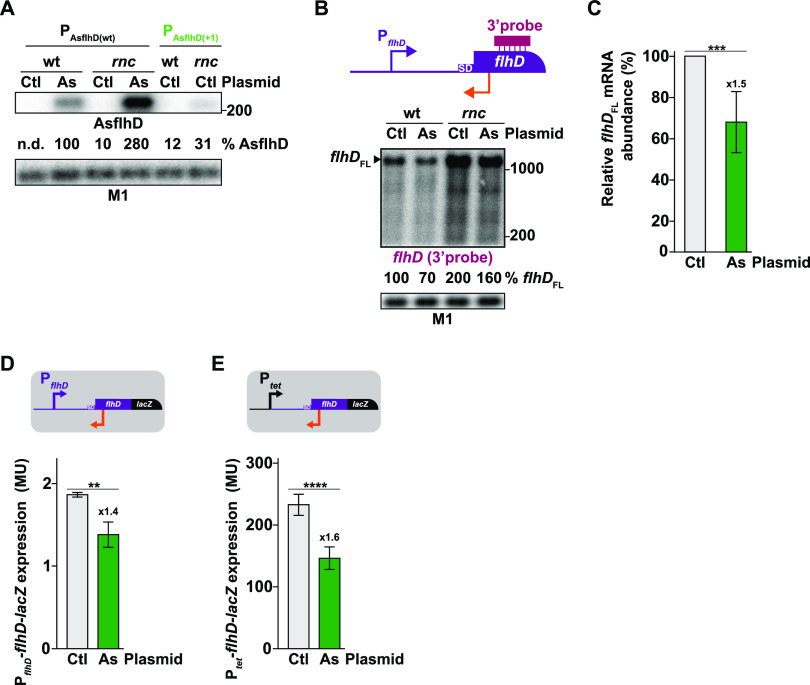
AsflhD represses the expression of flhD in *trans*. (A, B) The wt strain (MG1655-B) and its derivative containing the endogenous AsflhD promoter mutant (P_AsflhD(+1)_; ML241) and their *rnc* mutant derivatives (ML65 and ML341, respectively) containing the control pCA24N (Ctl) or the pCA24N AsflhD (As) plasmids were grown in the presence of 10^−4^ M isopropyl-β-d-thiogalactopyranoside (IPTG). Total RNA was extracted and subjected to northern blot analysis. The membranes were probed for (A) AsflhD and M1 RNA or (B) for *flhD* (using the 3′-probe as represented) and M1 RNA. (C) Average *flhD* mRNA abundance upon *trans*-overexpression of AsflhD (dark green), as shown in (B), was calculated as the mean of six biological replicates. (D, E) Expression of P*_flhD_*-*flhD*-*lacZ* (ML219) (D) and P*_tet_*-*flhD*-*lacZ* (ML233) (E) reporter fusions (gray) containing the plasmid pCA24N control (Ctl, in gray) or the plasmid pCA24N AsflhD (As, in dark green) was determined in the presence of 10^−4^ M IPTG. The values are the means of three biological replicates, and the bars indicate standard deviations. Statistical significance was determined by ANOVA. **, *P* ≤ 0.01; ***, *P* ≤ 0.001; ****, *P* ≤ 0.0001.

Increasing the endogenous AsflhD expression leads to the repression of *flhD* expression. Hence, we next investigated the ability of AsflhD to repress the expression of *flhD* when expressed in *trans*. AsflhD was overexpressed from a plasmid, under the control of a P*_tac_* promoter inducible by isopropyl-β-d-thiogalactopyranoside (IPTG). The short (242 nt) AsflhD is transcribed from the +1 to the +220 nucleotides (nt) relative to the TSS of AsflhD (i.e., +220 to +1 relative to the TSS of *flhD*) with a *rrnB*T2 terminator to stabilize the transcript. Under inducing conditions, *trans*-overexpression of AsflhD is stronger than *cis*-overexpression (9-fold in the *rnc* mutant; Fig. 7A) and can be directly observed in the wt strain. In agreement with results presented in the previous section (Fig. 6B), the *trans*-overexpression of AsflhD decreased the abundance of *flhD* mRNA both in the wt strain and in the *rnc* mutant (Fig. 7B and C). Furthermore, the short *flhD*_p_ RNA, accumulating in the *rnc* mutant upon *cis*-overexpression of AsflhD, was even more abundant upon *trans*-overexpression of AsflhD (Fig. S4B). It is not observed with the 3′-probe (Fig. 7B), confirming that it is derived from the region complementary to AsflhD. It is noteworthy that despite a higher expression in *trans*, plasmid-borne AsflhD is not as efficient at repressing the expression of *flhD*. This difference may be due to the presence of the *rrnB*T2 terminator and/or due to pervasive plasmid transcription, including transcripts antisense to the AsflhD insert, which can be observed when probing for the 5′-UTR of *flhD* in a strain deleted for the endogenous *flhD* locus (Fig. S4A). Of note, a similar problem has been reported for other vectors (both in eukaryote and bacteria) in which spurious expression of multiple overlapping transcripts was detected (18, 47).

10.1128/mbio.00981-22.5 FIG S4 *trans*-Overexpression of AsflhD represses *flhD* expression. (A, B) Total RNA was extracted from the wt (MG1655-B), its derivative containing a deletion of the *flhD* locus (Δ*flhD* in ML69) (A), its derivative containing the AsflhD promoter mutation (P_AsflhD(+1)_ in ML241) and their *rnc* derivatives (ML65, ML611 and ML341, respectively) containing the pCA24N control (Ctl) or AsflhD (As) grown in the presence of 10^−4^ M IPTG and subjected to northern blot analysis (B). The Membranes were probed for *flhD* (using the 5′-probe as represented on top) and M1 RNA. (C) Expression of P*_flhD_*-*flhD*-*lacZ* reporter fusions (gray; ML219) wt and its derivative containing the AsflhD promoter mutation (green; P_AsflhD(+1)_, ML241) containing the plasmid pCA24N control (light shaded bars; Ctl,) or the plasmid pCA24N AsflhD (dark shaded bars; As) was determined in the presence of 10^−4^ M IPTG. The values are the means of three biological replicates, and the bars indicate standard deviations. Statistical significance was determined by ANOVA. n.s., *P* ≥ 0.05; **, *P* ≤ 0.01; ****, *P* ≤ 0.0001. Download FIG S4, EPS file, 2.4 MB. Copyright © 2022 Lejars et al. 2022 Lejars et al. https://creativecommons.org/licenses/by/4.0/ This content is distributed under the terms of the Creative Commons Attribution 4.0 International license.

We confirmed these results on *flhD* mRNA levels by measuring the expression of the *flhD*-*lacZ* reporters under conditions of *trans*-overexpression of AsflhD and observed a small repression of *flhD* expression in both P*_flhD_*-*flhD*-*lacZ* and P*_tet_*-*flhD*-*lacZ* fusions (1.4 and 1.6-fold; Fig. 7D and E). It should be emphasized that these effects are independent of the *flhD* promoter (native P*_flhD_* or P*_tet_*). A combination of *cis-*overexpression (endogenous to the reporter) and *trans*-overexpression (from the plasmid) of AsflhD had little additive effect on the final repression when *flhD* was expressed from its own promoter (Fig. S4C), consistent with AsflhD repressing *flhD* expression by a common mechanism when expressed in *cis* or in *trans*. In summary, we show that *trans*-overexpression of AsflhD can repress *flhD* expression at both mRNA and translational levels.

### Control of the motility cascade by AsflhD.

**FIG 8:**
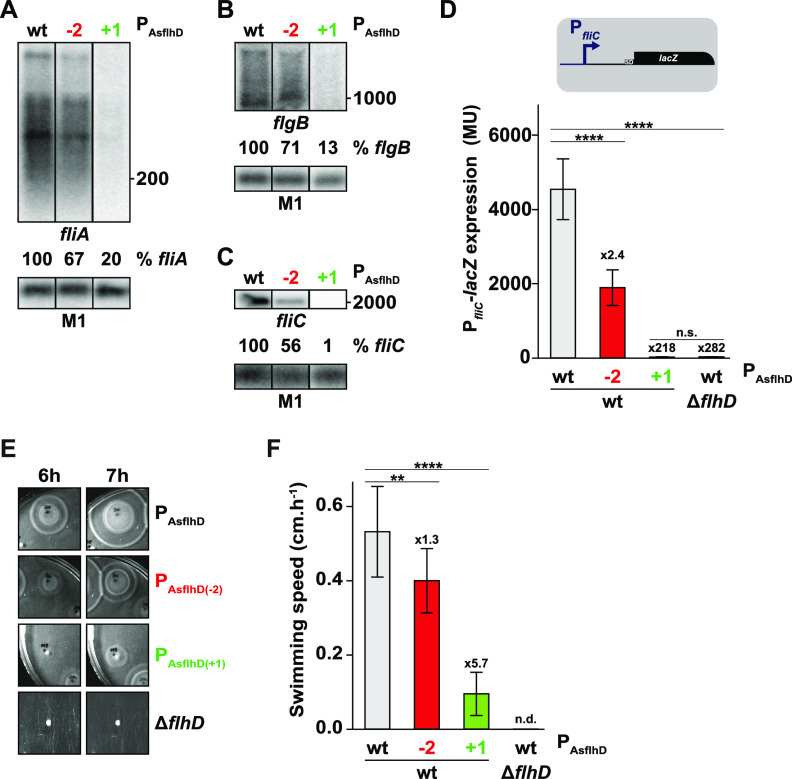
AsflhD controls the cascade of motility. (A to C) Total RNA was extracted from the wt (MG1655-B) and endogenous AsflhD promoter mutants (P_AsflhD(−2)_; ML73, P_AsflhD(+1)_; ML241) and subjected to northern blot analysis. The membranes were probed for *fliA* (A), *flgB* (B), and *fliC* and M1 RNA (C). The lanes were taken from the same membrane for each mRNA probed and uncropped membranes are shown in Text S1. (D) Expression of P*_fliC_*-*lacZ* (gray; ML616) reporter fusion and its derivative containing a deletion of the *flhD* locus (Δ*flhD*, ML621) or mutation of the AsflhD promoter (red; P_AsflhD(−2)_; ML617 and green; P_AsflhD(+1)_; ML615). (E) Representative plates showing effects of mutations reducing (P_AsflhD(−2)_) or increasing (P_AsflhD(+1)_) swimming motility compared to wt (MG1655-B) and loss of *flhD.* (F) Swimming motility speed, measured as described in the Materials and Methods section, are given for MG1655-B wt (gray) and its derivatives containing mutations reducing (P_AsflhD(−2)_; red) or increasing (P_AsflhD(+1)_; green) the expression of AsflhD. The values are means of seven (D) and three (F) biological replicates, and bars indicate standard deviations. Statistical significance was determined by ANOVA. **, *P* ≤ 0.01; ****, *P* ≤ 0.0001. n.s., not significant.

The *flhDC* operon encodes the FlhD_4_C_2_ transcriptional regulator, main activator of the cascade of motility-related genes, which are divided into three classes (48). The *flhDC* operon encodes the only class I protein complex, FlhD_4_C_2_, which is essential for expression of class II genes, which in turn control class III genes. Thus, we next investigated the impact of changing AsflhD levels on representative class II and III genes. We selected the following class II genes: *fliA* that encodes FliA, the sigma factor for class III motility genes, and *flgB* that encodes FlgB, a component of the flagella proximal rod and the class III gene *fliC* gene, encoding the main component of flagella, FliC. The amounts of *fliA*, *flgB*, and *fliC* mRNAs are reduced (from 1.2- to 1.9-fold) when AsflhD expression is reduced (mutations P_AsflhD(−2),_ P_AsflhD(−1)_, and P_AsflhD(−3)_) and strongly reduced (from 5- to 100-fold) upon *cis*-overexpression of AsflhD (P_AsflhD(+1)_; Fig. 8A to C; Fig. S5A to C). The effects were strongest for the class III gene *fliC*. Using a P*_fliC_*-*lacZ* transcriptional reporter fusion, we confirmed the reduced expression of *fliC* when AsflhD levels decreased (from 1.6- to 2.4-fold; Fig. 8D; Fig. S5D). Surprisingly but consistent with the northern blot (Fig. 8C; Fig. S5C), *cis*-overexpression of AsflhD by the P_AsflhD(+1)_ mutation produced a very large decrease in *fliC-lacZ* expression (218-fold; Fig. 8D). It has previously been observed that a modest reduction of *flhD* transcription could lead to a strong repression of the motility cascade (49, 50), and this could be the case for *fliC*, which depends upon the FlhD_4_C_2_-dependent class II sigma factor, FliA, for its expression. We had measured a reduction in *flhD* expression and mRNA and a greater repression of class II gene *fliA*, but the very strong repression of *fliC-lacZ* exerted by the *cis*-overexpression of AsflhD raised the question of whether the P_AsflhD(+1)_ mutation affected the activity of FlhD since it leads to the mutation of the 12th amino acid (FlhD_D12N_). Thus, we cannot exclude that this change might affect FlhD activity. However, it was previously shown that the FlhD_D12A_ mutation did not affect motility (51), and D12 is not in a region involved in contacting FlhC in the FlhD_4_C_2_ complex (52). Moreover, several pieces of evidence show that the FlhD_D12N_ (P_AsflhD(+1)_) protein is still active, in particular because the P_AsflhD(+1)_ mutant strain is still motile (see below).

10.1128/mbio.00981-22.6 FIG S5 AsflhD silencing represses the induction of the motility cascade. (A to C) Total RNA was extracted from the wt (MG1655-B) and endogenous AsflhD promoter mutants (P_AsflhD(−2)_ in ML73, P_AsflhD(−1)_ in ML609, and P_AsflhD(−3)_ in ML610) and subjected to northern blot analysis. The membranes were probed for *fliA* (A), *flgB* (B), *fliC* (C), and M1 RNA. (D) Expression of P*_fliC_*-*lacZ* (gray; ML616) reporter fusion and its derivative containing a deletion of the *flhD* locus (Δ*flhD*, ML620) or mutation of the AsflhD promoter (red; P_AsflhD(−2),_ ML617; P_AsflhD(−1)_, ML618; and P_AsflhD(−3)_, ML619). The values are means of 10 biological replicates, and the bars indicate standard deviations. Statistical significance was determined by ANOVA. ****, *P* ≤ 0.0001. Download FIG S5, EPS file, 2.6 MB. Copyright © 2022 Lejars et al. 2022 Lejars et al. https://creativecommons.org/licenses/by/4.0/ This content is distributed under the terms of the Creative Commons Attribution 4.0 International license.

To verify that the changes in AsflhD gene expression were reflected in bacterial behavior, we analyzed the effect of AsflhD on motility using low-agar plates and observed a reduction in the swimming speed when AsflhD expression was either reduced (1.3-fold in P_AsflhD(−2)_) or increased (5.7-fold in P_AsflhD(+1)_), while a strain deleted for the *flhD* locus (Δ*flhD*) showed no motility (Fig. 8E and F). Swimming motility was measured in super optimal broth in the presence of magnesium (SOB+Mg). This medium (as opposed to LB used for other studies) was preferred to allow measurements within 12 h, since *flhD* expression and motility are increased (see Materials and Methods). In addition, we verified that expression of the P*_flhD_*-*flhD*-*lacZ* and P*_flic_*-*lacZ* reporter fusions were also repressed by the *cis*-overexpression of AsflhD (P_AsflhD(+1)_) in SOB+Mg (5.7- and 81-fold, respectively; Fig. S6). Importantly in SOB+Mg the expression of *fliC-lacZ* in the P_AsflhD(+1)_ mutant is 15-fold higher than in a strain deleted for *flhD* (Fig. S6B), confirming that the FlhD_(D12N)_ protein is at least still partially functional.

10.1128/mbio.00981-22.7 FIG S6 Control of the motility by AsflhD cascade in SOB+Mg. (A) Expression of P*_flhD_*-*flhD*-*lacZ* reporter fusions (gray; ML219) wt and its derivative containing the AsflhD promoter mutation (green; P_AsflhD(+1)_, ML241) and (B) expression of P*_fliC_*-*lacZ* (gray; ML616) reporter fusion and its derivative containing a mutation of the AsflhD promoter (green; P_AsflhD(+1)_, ML615) or deletion of the *flhD* locus (black; Δ*flhD*, ML620) were analyzed in Super optimal broth in the presence of 2.4 g/liter MgSO_4_ (SOB+Mg) at *A*_600_ ≈ 0.4. The values are the means of four biological replicates, and the bars indicate standard deviations. Statistical significance was determined by ANOVA. ****, *P* ≤ 0.0001. Download FIG S6, EPS file, 1.4 MB. Copyright © 2022 Lejars et al. 2022 Lejars et al. https://creativecommons.org/licenses/by/4.0/ This content is distributed under the terms of the Creative Commons Attribution 4.0 International license.

We also attempted to confirm that AsflhD expressed in *trans* from the plasmid reduced *fliC* expression. As shown above, the *trans*-overexpression of AsflhD modestly represses the expression of *flhD* (Fig. 7B to E), while we found a small reduction of *fliC* expression at both mRNA (1.6-fold; Fig. S7A) and translational levels (2-fold; Fig. S7B) compared to much stronger effects on *fliC* by the P_AsflhD(+1)_ mutation. Expression of AsflhD in *trans* did, however, lead to a slight reduction of the swimming speed (1.2-fold; Fig. S7D).

10.1128/mbio.00981-22.8 FIG S7 *trans*-Overexpression of AsflhD represses motility. (A) Total RNA was extracted from the wt (MG1655-B) containing the pCA24N control (Ctl) or AsflhD (As) and subjected to northern blot analysis. The membrane was probed for *fliC* and M1 RNA. (B, C) Expression of P*_fliC_*-*lacZ* (gray) reporter fusion containing the pCA24N control (gray; Ctl) or pCA24N AsflhD (dark green; As) in the wt strain (ML616) (B) or in the strain deleted for *flhD* (*ΔflhD*, ML621) (C). (D) Representative plates showing swimming motility in wt bacteria containing the pCA24N control (gray; Ctl) or AsflhD (dark green; As). (E) Swimming motility speeds, measured as described in the Materials and Methods section, are the means of at least three biological replicates, and the bars indiate standard deviations. Statistical significance was determined by ANOVA. n.s., *P* ≥ 0.05; **, *P* ≤ 0.01; ****, *P* ≤ 0.0001. Download FIG S7, EPS file, 2.7 MB. Copyright © 2022 Lejars et al. 2022 Lejars et al. https://creativecommons.org/licenses/by/4.0/ This content is distributed under the terms of the Creative Commons Attribution 4.0 International license.

It is possible that the effect of AsflhD could also be partially due to independent regulatory events on other targets within the motility cascade. However, a bioinformatics search (TargetRNA2 [53] and CopraRNA [54]) for possible direct *trans* targets of AsflhD found no candidates among genes from the motility cascade. Furthermore, as shown previously (48) and above (in SOB+Mg; Fig. S7C), *fliC* expression is dependent upon *flhD* via the sigma factor *fliA*. *trans*-overexpression of AsflhD had no effect on the expression of *fliC-lacZ* in a *flhD* mutant strain (Fig. S7C). Thus, all results are congruent with the notion that AsflhD affects the expression of *fliC* and motility through the repression of *flhD* (Fig. 8; Fig. S7D). In summary, both reduced and increased expression of AsflhD repress the expression of *flhD*, which in turn leads to repression of the whole cascade of motility and a reduction of the swimming speed.

### Transcriptional repression of *flhD* by AsflhD *in vitro*.

**FIG 9:**
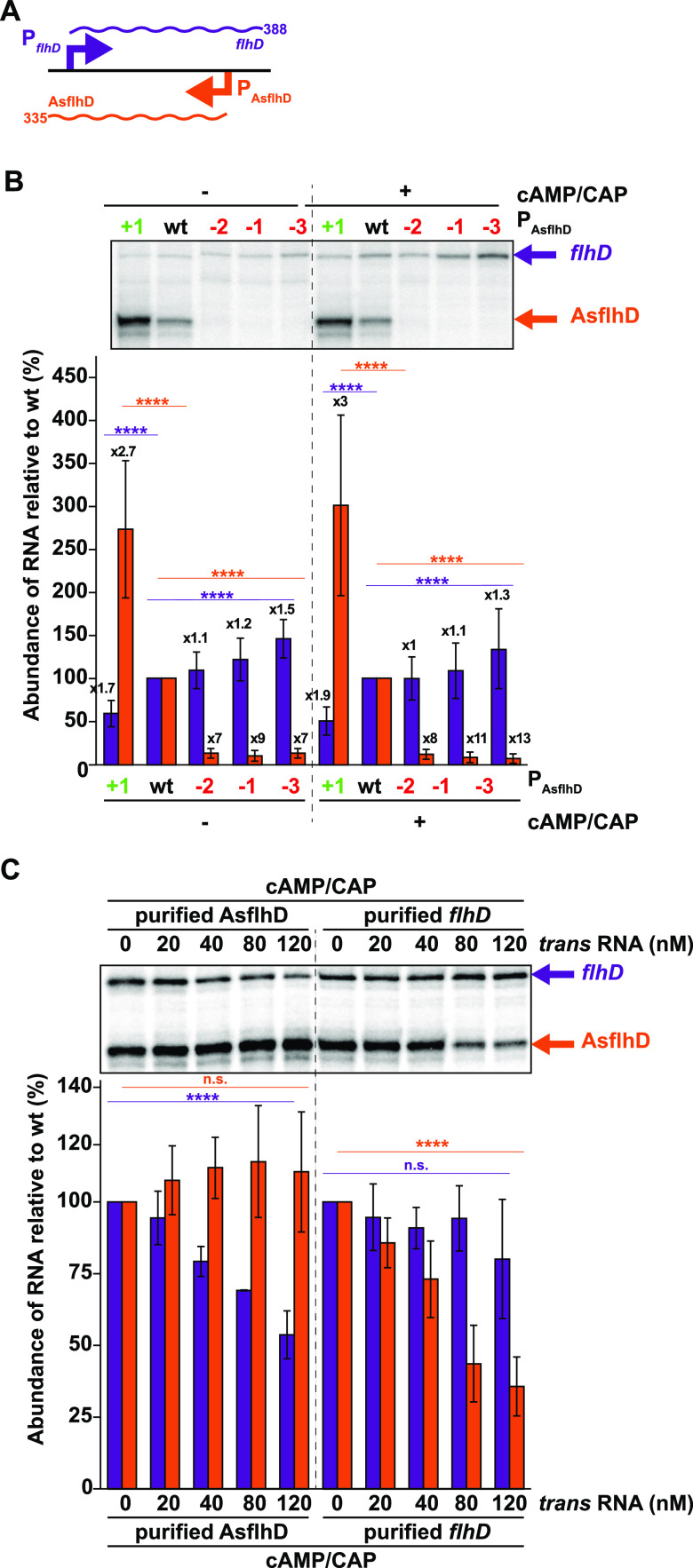
AsflhD represses the transcription of *flhD in vitro*. (A) Schematic representation of the template used for the *in vitro* transcription assay carrying the P*_flhD_* promoter driving the expression of a 388-nt transcript (purple) and the P_AsflhD_ promoter driving the expression of a 335-nt transcript (orange). The linear DNA template was constructed using the oligonucleotides LM191 and LM9 (Table S1) and corresponds to −76 to +388 of the *flhD* transcript relative to its TSS, with a 40-nt extension carrying the *rrnB*T2 terminator (fragment length, 504 bp). This fragment carries the native *flhD* promoter (−10 and −35 sites) and includes the cAMP/CAP site at −72 compared to the *flhD* TSS, at its upstream extremity. (B, C) *In vitro* transcription assays were performed on templates carrying wt, P_AsflhD(+1)_, P_AsflhD(−2)_, P_AsflhD(−1)_, and P_AsflhD(−3)_ mutations as described in the supplemental Materials and Methods section (Text S1), with or without the addition of 100 nM CAP and 0.2 mM cAMP for 15 min at 37°C before addition of RNA polymerase (RNAP) (B) or with 100 nM CAP and 0.2 mM cAMP and the addition of *in vitro* purified AsflhD or *flhD* transcripts (C) to the reaction at the indicated concentrations before addition of RNAP. The samples were analyzed on sequencing gels. The relative intensities of the indicated bands (*flhD* in purple and AsflhD in orange) were analyzed. The values are means of four (B) and three (C) replicates, and the bars indicate standard deviations. Statistical significance was determined by ANOVA and is indicated for either AsflhD (in purple) or *flhD* (in orange) RNA. n.s., *P* ≥ 0.05; ****, *P* ≤ 0.0001.

We next investigated the mechanism of action of AsflhD. The overexpression of AsflhD represses the expression of *flhD* at the mRNA and translation level without affecting the stability of the *flhD* mRNA. Hence, we reasoned that AsflhD could be involved in the transcriptional repression of *flhD*. To test this hypothesis, we performed *in vitro* transcription experiments using a DNA template corresponding to the *flhD* gene from 76 nt before to 388 nt after the TSS of *flhD*, which allows the transcription of a 388-nt *flhD* RNA (*flhD*_FL_) and of a 335-nt AsflhD RNA (AsflhD) (Fig. 9A). We compared the abundance of both transcripts synthesized from the latter DNA fragment to those generated from templates carrying the promoter mutations, leading to either decreased (P_AsflhD(−2),_ P_AsflhD(−1)_, and P_AsflhD(−3)_) or increased expression (P_AsflhD(+1)_) of AsflhD. We confirmed that expression of AsflhD is strongly impaired when transcribed from the template carrying mutations reducing AsflhD repression (around 10-fold in P_AsflhD(−2)_, P_AsflhD(−1)_, and P_AsflhD(−3)_), and AsflhD expression increases from the template carrying the mutation enhancing the expression of AsflhD (2.7-fold in P_AsflhD(+1)_; Fig. 9B, left panel, orange bars). At the same time, AsflhD overexpression resulted in a decrease in the transcription of *flhD* RNA (1.7-fold in P_AsflhD(+1)_), while the loss of AsflhD led to an increase of the transcription of *flhD* RNA (1.1-fold in P_AsflhD(−2)_, 1.2-fold in P_AsflhD(−1)_, and 1.5-fold in P_AsflhD(−3)_; Fig. 9B, left panel, purple bars).

The transcription factor CAP promotes the transcription of *flhD* by binding to a sequence located 72 nt upstream from the TSS of *flhD* (55). As expected, the presence of cAMP/CAP increased the transcription of *flhD*, which was still reduced when expression of AsflhD was increased (1.9-fold) but only slightly increased when AsflhD expression was reduced from the down-mutations (maximum 1.3-fold for P_AsflhD(−3)_; Fig. 9B, right panel, purple bars). *In vitro* transcription assays were performed in a single round of elongation in the presence of heparin and with RNAP prebound to templates; hence, the observed effects are restricted to the elongation step and should be independent of the initiation of transcription.

We also investigated the effect of AsflhD on *flhD* transcription using a template in which the P*_tet_* promoter replaced the P*_flhD_* promoter (Fig. S8A). This template produces the same 388-nt *flhD* RNA but a shorter (260 nt) AsflhD RNA. Using these templates with the strong P*_tet_* promoter, we observed only a slight reduction of *flhD* mRNA accumulation from the template carrying the P_AsflhD(+1)_ up- or P_AsflhD_ down-mutations (maximum 1.2-fold; Fig. S8B, purple bars). In summary, *in vitro* enhanced expression of AsflhD from the P_AsflhD(+1)_ fragment leads to the repression of transcription elongation of *flhD* from the P*_flhD_* in the presence or absence of CAP/AMPc, while reduction in AsflhD expression has only a modest positive effect on *flhD* expression.

10.1128/mbio.00981-22.9 FIG S8 AsflhD repression of the transcription of flhD is independent of the *P_flhD_* promoter. (A) Schematic representation of the template used for the *in vitro* transcription assay carrying the P*_tet-_flhD* promoter driving the expression of a-388 nt transcript (purple) and the P_AsflhD_ promoter driving the expression of a 260-nt transcript (orange). The DNA templates were constructed using the LM213 and LM9 oligonucleotides and carry chromosomal sequences starting at the *flhD* TSS with a 40-nt extension carrying the tetracycline promoter (36) so that P*_tet_* transcription starts at the position of the *flhD* TSS. (B, C) *In vitro* transcription assays were performed on templates carrying wt, P_AsflhD(+1)_, P_AsflhD(−2)_, P_AsflhD(−1)_, and P_AsflhD(−3)_ mutations (B) and on the wt template after addition of *in vitro* synthesized AsflhD or *flhD* transcripts before addition of the RNAP to the reaction at the indicated concentrations (C) (see supplemental Materials and Methods section in Text S1). The relative intensities of the indicated bands (*flhD* in purple and AsflhD in orange) were quantified as described in the supplemental Materials and Methods section (Text S1) from four (B) and three (C) replicates. The values are shown as means, and the bars indicate standard deviations. Statistical significance was determined by ANOVA and is indicated for either AsflhD (in purple) or *flhD* (in orange) RNA. n.s., *P* ≥ 0.05; **, *P* ≤ 0.01; ***, *P* ≤ 0.001; ****, *P* ≤ 0.0001. Download FIG S8, EPS file, 2.0 MB. Copyright © 2022 Lejars et al. 2022 Lejars et al. https://creativecommons.org/licenses/by/4.0/ This content is distributed under the terms of the Creative Commons Attribution 4.0 International license.

### Mutual repression of *flhD* and AsflhD transcription in *trans*.

We also investigated the effect of including purified AsflhD or *flhD* RNA on the transcription of both *flhD* and AsflhD using the same linear DNA templates. The addition of increasing amounts of AsflhD led to a linear decrease of *flhD* (up to 1.9-fold in the presence of 120 nM AsflhD; Fig. 9C) without affecting the level of AsflhD expression. The reciprocal assay (addition of increasing concentrations of purified *flhD* RNA) led to a linear decrease of the amount of AsflhD synthesized (up to 2.8-fold in the presence of 120 nM *flhD*), while the amount of *flhD* was not affected. We performed the same assay with the template carrying the P*_tet_* promoter (Fig. S8A) and observed similar results (Fig. S8C).

In summary, AsflhD can repress the transcription elongation of *flhD* both in *cis* and in *trans*. Thus, we propose that AsflhD asRNA and *flhD* mRNA are involved in their mutual transcriptional attenuation in which the interaction of one molecule with the other leads to a reduction in transcription at the level of transcription elongation (see discussion).

## DISCUSSION

**FIG 10:**
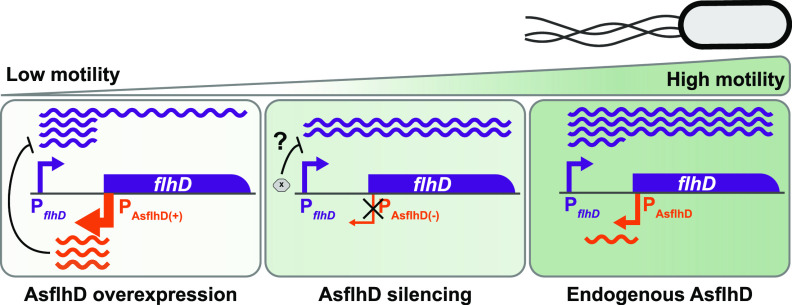
Schematic representation of the regulatory function of AsflhD. Swimming motility is slightly reduced *in vivo* when the expression of AsflhD (orange) is reduced (middle panel) *via* the decreased transcription of *flhD* mRNA (purple) possibly involving another factor (represented in gray). Upon overexpression (left panel), AsflhD represses directly the transcription of *flhD* mRNA both *in vitro* and *in vivo*, mainly via transcriptional attenuation leading to a strong decrease of swimming motility.

Regulatory RNA molecules are often part of complex genetic networks in bacteria. They correspond to a heterogeneous class of molecules that differ in gene organization, size, and function. Our goal was to detect, identify, and investigate the function of antisense transcripts in E. coli. In this work, we provide evidence that asRNAs can be important players in the expression of transcriptional factors despite their low level of expression. In particular, we have shown that changes in the level of the asRNA to *flhD* can affect the expression of its target and lead to defects in swimming motility as recapitulated in Fig. 10.

### Conservation of AsflhD and AsphoP.

Sequences of intergenic regions are usually less well conserved than their neighboring coding sequences, allowing the rapid evolution of regulatory signals. This characteristic is reflected in the presence and conservation of promoters for asRNA and sRNAs. For asRNA initiated within the coding sequence of their target, nucleotide changes within the coding region risk upsetting the function of the ORF and could be counterselected. AsflhD corresponds almost entirely to the 5′-UTR of *flhD* but with the promoter located in the ORF, which is fairly well conserved in enterobacteria (Fig. 3A) (51). Thus, the conservation of the promoter of AsflhD could be the result of direct selection for FlhD activity or for the regulatory function of AsflhD (potentially) controlling the expression of FlhD in these bacteria. In S. enterica serovar Typhi, AsfD, a long asRNA complementary to *flhDC* and *motA* mRNAs, was observed as a 2,000-nt fragment by northern blotting in a wt strain (56). AsfD was implicated in the positive regulation of *flhDC* during stationary phase by an uncharacterized mechanism. However, this asRNA is likely to originate from a region located downstream from the *flhDC* locus that is not present in E. coli K-12 MG1655 (and no equivalent transcript was observed in our and independent transcriptomic data sets). To our knowledge, no transcriptomic data set is available in Salmonella species inactivated for RNase III. However, a low abundance asRNA to *flhD*, whose start corresponds to that of AsflhD and that was slightly enriched under nitric-oxide shock, has been previously detected (http://bioinf.gen.tcd.ie/cgi-bin/salcom.pl?_HL) (57). Its localization is consistent with the conservation of AsflhD in S. enterica but we cannot exclude that this transcript is processed from AsfD in S. enterica.

In the case of AsphoP, the promoter also located in the coding region of the *phoP* ORF is not conserved in other enterobacteria. The lack of conservation in other bacteria suggests that, in contrast to AsflhD, any function of AsphoP may be unique to E. coli, where it most likely arose.

### Role of RNase III in the degradation of AsflhD and *flhD*.

We showed that RNase III can cleave both AsflhD and *flhD* and degrade the AsflhD-*flhD* RNA duplex *in vitro* (Fig. S2B to D). In addition, RNase III affects the expression and stability of *flhD* independent of AsflhD expression *in vivo* (Fig. S1; Fig. 6A). Hence, there appear to be two aspects to its action: first, the complete degradation of AsflhD-*flhD* RNA duplexes, and second, the destabilization of *flhD* mRNA by cleavages within its 5′-UTR. Degradation of *flhD*/AsflhD duplexes appears to be a stoichiometric event, removing *flhD* proportionally to the level of AsflhD transcription, since AsflhD is never detected free when RNase III is active in the wt strain. The second mechanism presumably involves cleavages within the 5′-UTR that likely modify the complex post-transcriptional regulation of *flhD* and may facilitate RNase E-mediated degradation.

### Mechanism of regulation by AsflhD.

Transcriptional repression by asRNAs in bacteria has been proposed to arise either from transcriptional interference (upon colliding convergent RNAP) or from transcriptional attenuation (upon binding of the asRNA to its complementary target) (58). For example, the asRNA RNAβ promotes the premature termination of the operon *fatDCBA-angRT* in Vibrio anguillarum (59), while the asRNA anti-Q in Enterococcus faecalis is responsible for both transcriptional interference due to RNAPs collisions and attenuation by an uncharacterized mechanism (60). In this work, we show that both a decrease and an increase in AsflhD expression reduce the abundance of *flhD* mRNA and *flhD* translation *in vivo* (Fig. 6A to D) without significantly affecting the stability of the *flhD* mRNA (Fig. S1). Using an *in vitro* system, we reveal that AsflhD synthesized *in situ* or added exogenously can repress the transcription elongation of *flhD* (Fig. 9; Fig. S8). Since exogenous AsflhD can repress the transcription of *flhD in vitro* to a similar extent as when it is synthesized in *cis* from its endogenous promoter (up to 2-fold repression; Fig. 9B and C; Fig. S8B to C), we propose that AsflhD represses the transcription elongation of *flhD* mainly via transcriptional attenuation. Furthermore, our experiments do not detect the accumulation of a shorter transcript *in vitro* upon addition of one or the other of the transcripts, suggesting that binding of AsflhD to *flhD* does not stabilize a terminator structure but could rather modify the stability of the elongating RNAP, leading to heterogenous 3′-termini as observed for AsflhD *in vivo* by cRT-PCR (Fig. 4A).

The repression of *flhD* expression is weaker (1.4-fold compared to 4.9-fold) when AsflhD is expressed in *trans* compared to in *cis* in the P*_flhD_*-*lacZ* reporter fusion (Fig. 6D and E and 7D and E). This suggests that AsflhD could also repress *flhD* by transcriptional interference when expressed from its own promoter. However, we cannot rule out that these variations are due to differences in the stoichiometry between *flhD* and AsflhD RNAs. In addition, the transcription of AsflhD from the same locus as *flhD* could lead to the increased local concentration of AsflhD in the vicinity of the nascent *flhD* transcript, thus enabling AsflhD to interfere with and terminate *flhD* transcription more efficiently.

Experiments performed *in vitro* provide evidence for the transcriptional attenuation of *flhD* expression upon overexpression of AsflhD. However, we also observed that a reduction in AsflhD expression leads to a decrease in *flhD* expression *in vivo*. This phenomenon was not observed *in vitro*, so it is likely that this second positive regulatory mechanism involves other factors, such as the numerous post-transcriptional regulators (RNA-binding chaperones and sRNAs) of *flhD* expression (35). Close to the translational start of *flhD*, binding of the McaS sRNA is required to expose the ribosome-binding site and activate translation (61), while the RNA-binding chaperone CsrA protects the 5′-end of *flhD* (45). On the contrary, binding of the sRNAs, OxyS, ArcZ, OmrA, and OmrB represses translation (35). AsflhD binding could interfere with the binding of any of these sRNAs at their sites along the 5′-UTR of the *flhD* mRNA. Remarkably, most post-transcriptional regulatory events on *flhD* were shown to have weak effects (i.e., often close to 2-fold repression [35] or activation [45, 61]). Hence, deciphering the effect of each regulator and its interference with the regulation by AsflhD will be an interesting challenge for future studies.

### Outlook.

In this work, we have demonstrated the existence of asRNAs complementary to four major regulators of gene expression in E. coli. As we have shown for the asRNA AsflhD, it is likely that they affect both the expression of their direct target and the downstream control of the target’s regulon. Regulatory RNAs are far from being fully understood in bacteria, and new mechanisms of action are likely to be discovered. Furthermore, as in the case of AsflhD, asRNAs demonstrate unexpected regulatory functions that raise the question as to how, when, and to what extent asRNAs participate in complex regulatory circuits.

## MATERIALS AND METHODS

### Bacterial strains and culture conditions.

Strains and plasmids used in this work are listed in Table S1. Constructions and mutations were made by using primers given in Table S1 and are described in the supplemental Materials and Methods (Text S1). Strains were grown in LB medium at 37 or 30°C and shifted to 42 or 46°C for the heat-shock experiments, and samples were taken in the mid-log phase (*A*_600_ ≈ 0.4) or as indicated. Strains carrying the pCA24N control and pCA24N AsflhD (containing the 220 first nt of AsflhD relative to its TSS followed by the *rrnB* T2 terminator) were grown in the presence of chloramphenicol and induced by isopropyl β-d-1-thiogalactopyranoside (10^−4^ M).

### Northern blotting and RNA methods.

Total RNA was extracted using the hot-phenol procedure (62). Five μg of total RNA were electrophoresed either on 1% agarose with 1× Tris-borate-EDTA (TBE) or 6% polyacrylamide gels (19/1) with 7 M urea and 1× TBE for analysis by northern blotting (63, 64) along with a RiboRuler High-Range marker (ThermoFisher) or radiolabeled MspI-digested pBR322 (NEB). The membranes were hybridized with cRNA probes. DNA templates for the synthesis of the RNA probes were obtained by PCR amplification using the pair of “m” and “T7” oligonucleotides (Table S1). The probes were synthesized by T7 RNAP with [α-^32^P]UTP yielding uniformly labeled RNAs (65). The membranes were also probed with M1 RNA (or 5S) as loading control by using 5′-end-labeled primers (Table S1). DNA templates for *in vitro* processing and *in vitro* transcription assay carrying a T7 promoter sequence were generated by PCR using primers Up-T7-flhD + 308/Down-flhD and Up-T7-AsflhD/Down-AsflhD (Table S1). They allow the transcription of the first 308 nt of *flhD* and of the first 256 nt of AsflhD, respectively. RNAs were synthesized by T7 RNAP with [α-^32^P]UTP as a tracer and were gel purified. Our previously published transcriptomic data set (available in the ArrayExpress database at EMBL-EBI under accession number E-MTAB-9507) (23) was used to compare the transcriptomes of the wild-type (N3433) and the RNase III-deficient strain (IBPC633) and sorted against independently analyzed data sets (described in Table 1) in which asRNAs and TSSs have been identified.

### β-Galactosidase assays.

Reporter fusions were constructed in the *lacZ* locus as described in the supplemental Materials and Methods (Text S1). In brief, the P_AsphoP_-*lacZ* fusion contains nt −150 to +15 from the AsphoP TSS, the P_AsflhD_-*lacZ* fusion contains nt −165 to +15 relative to the AsflhD TSS, the P*_flhD_*-*lacZ* fusion contains nt −108 to +300 from the *flhD* TSS, and the fusion P*_tet_*-*flhD*-*lacZ* contains the P*_tet_* promoter sequence followed by nt +1 to +300 relative to the *flhD* TSS. The *fliC-lacZ* fusion was described previously (66). It carries nt −79 to +96 with respect to the *fliC* TSS. The cultures were initiated at *A*_600_ = 0.02 and sampled at *A*_600_ = 0.4 to 0.5. Samples (100 or 200 μL) were lysed in Z buffer (1 mL total). β-Galactosidase activity was assayed as described (67); the results are the means of at least three biological replicates as indicated in the legends. Since the *lacZ* mRNA was previously reported to be negatively regulated by RNase III through multiple cleavages within the *lacZ* mRNA ORF (68, 69), we have not attempted to compare *lacZ* reporter fusion expression between wt and *rnc* mutant.

### Circular RT-PCR.

Circular RT-PCR was performed with total RNA extracted from N3433 and IBPC633 treated with 5′-polyphosphatase. After circularization with T4 RNA ligase 1 (Biolabs), mflhD2 was used to prime reverse transcription and mflhD6 and masflhD10 to generate PCR products (Table S1), which were cloned and analyzed as described (70). It should be noted that the efficiency of ligation by the RNA ligase 1 was previously shown to be affected by the presence of secondary structures, which could explain the exclusion of double-stranded RNAs (43).

### RNA band-shift assay and *in vitro* processing by RNase III.

AsflhD (256 nt) and *flhD* (308 nt) RNAs were synthesized as described in the section “Northern blotting and RNA methods.” Transcript 5′-end labeling, hybridization, RNase III digestion, and sample analysis were performed as described previously (70, 71) and are also described in the supplemental Materials and Methods (Text S1).

### *In vitro* transcription assay.

Single-round *in vitro* transcription experiments were carried out on linear templates as described in the supplemental Materials and Methods (Text S1). AsflhD and *flhD* RNAs added in *trans* were synthesized as described in the section “Northern blotting and RNA methods.”

### Motility assay.

Stationary-phase bacterial cultures (wt, MG1655-B; P_AsflhD(−2)_, ML73; and P_AsflhD(+1)_, ML241), with or without the pCA24N control (Ctl) or the pCA24N AsflhD (As) plasmid, were inoculated (2 μL) on soft agar (0.2 g/liter) Super optimal broth motility plates (containing 2.4 g/liter MgSO_4_ and 10^−4^ M IPTG for strains carrying pCA24N plasmids) at 37°C and pictures were taken using a Gel Doc (Bio-Rad) imager between the beginning and the end of the linear swimming motility period (from 5 to 8 h). Representative images of swimming motility are shown at 6 and 7 h. Super optimal broth was used because bacteria are more motile (presumably due to its lower NaCl concentration, which alleviates OmpR repression of *flhD* [72, 73]), which allows accurate measurements within 12 h. Swimming speed was then calculated by comparing the increase of motility diameters over time.

### Data availability.

The RNA-seq data set comparing wt and *rnc* mutant (RNase III inactivation) is available in the ArrayExpress database at EMBL-EBI under accession number E-MTAB-9507.
