# Supplementary material for: Regulatory Interplay between RNase III and Antisense RNAs in E. coli: the Case of AsflhD and FlhD, Component of the Master Regulator of Motility
Source: mBio. 2022 Aug 24;13(5):e00981-22. doi: 10.1128/mbio.00981-22 (PMC9600491; doi:10.1128/mbio.00981-22)
Supplement: TEXT S1 [file mbio.00981-22-s0009.docx]

***Text S1: Supplemental materials and methods and uncropped northern blots***

*Bacterial strain construction*

To construct endogenous mutants of AsflhD promoter, a *cat*-*sacB* cassette was first introduced in the *flhD* locus by recombineering using primers 5’-cat-sacB and 3’-cat-sacB following previously described λ red-based procedures (1), and then replaced by recombineering using a DNA fragment containing mutations introduced by a two-step PCR using primer Up-cat-sacB and mutated primers Down-PAsflhD-2, Down-PAsflhD-1, Down-PAsflhD-3 or Down-PAsflhD+1 (Table S2). Recombinant clones were selected on LB plates without NaCl, supplemented with 6% sucrose. Chloramphenicol-sensitive colonies were subsequently purified. The mutant versions of the sequences of the promoters of AsflhD were designed in order to minimally affect the amino acid sequence of the proteins encoded on the opposite direction and not to introduce rare codons. In P_AsflhD(-2)_, P_AsflhD(-1)_ and P_AsflhD(-3)_ no amino acid change is introduced and in P_AsflhD(+1)_ the 12^th^ residue, aspartic acid, is replaced by an asparagine (D12N).

To construct *lacZ* reporter fusions of AsphoP, DNA fragments corresponding to nts −150 to +15 of AsphoP (relative to its TSS) were amplified by PCR using Phusion high-fidelity DNA polymerase (NEB) with primers Up-PAsphoP and Down-PAsphoP on genomic DNA of strain MG1655-B. The fragment for recombineering of the mutated AsphoP was made by a 2-step PCR using first primers Up-PAsphoP and Down-PAsphoP- and second Down-PAsphoP and Up-PAsphoP- (Table S2).

To construct *lacZ* reporter fusions of AsflhD, DNA fragments corresponding to nts -165 to +15 of AsflhD (relative to its TSS) were amplified by PCR using Phusion high-fidelity DNA polymerase (NEB) with primers Up-PAsflhD-165 and Down-PAsflhD+15 on the genomic DNA of strains MG1655-B (P_AsflhD(wt)_), ML73 (P_AsflhD(-2)_), ML609 (P_AsflhD(-1)_), ML610 (P_AsflhD(-3)_) and ML241 (P_AsflhD(+1)_).

To construct *lacZ* reporter fusions of *flhD*, DNA fragments corresponding to nts -108 to +300 (P*_flhD_*-*lacZ*) and +1 to +300 (P*_tet_*-*flhD*-*lacZ*) of *flhD* (relative to its TSS) were amplified by PCR using Phusion high-fidelity DNA polymerase (NEB) with primers Up-PflhD-108 or Up-Ptet-flhD+1 and Down-PflhD+300 on the genomic DNA of strains MG1655-B (P_AsflhD(wt)_), ML73 (P_AsflhD(-2)_) and ML241 (P_AsflhD(+1)_).

In addition, primers contain in their 5’-end homologous sequence to the *rrnB* T2 terminator and to *lacZ* from the nt -17 (for transcriptional AsphoP and AsflhD fusions) or from the nt +28 (for *flhD* fusions, primers Up-PflhD-108 or Up-Ptet-flhD+1 and Down-PflhD+300) relative to the translation start of *lacZ*.

The final PCR products were transformed and recombined in the MG2114 strain (as described above) to replace the *cat-sacB* cassette of a chromosomal *rrnB*T2-*cat*-*sacB*-*lacZ* construct at the *lacZ* locus.

Derivative strains containing the reporter fusions were constructed by P1 transduction.

The pCA24N AsflhD plasmid (Table S1) was constructed to overexpress AsflhD transcript from the P*_tac_* promoter by amplification of AsflhD from the +1 to the +220 nts followed by the 35 nts *rrnBT2* terminator with primers pCA24N AsflhD+1 and AsflhD +220-rrnBT2 on the genomic DNA of strain MG1655-B (Table S2). The upstream Xho I and downstream Hind III restriction sites were then added by a 2-step PCR using primers pCA24N-Xho1-AsflhD+1 and pCA24N-HindIII-AsflhD+220 (Table S2). The fragment was inserted into pCA24N vector digested with *Xho*I and *Hind*III using T4 DNA ligase (NEB). The ligation product was then transformed in super-competent DH5- α cells (C2992H, NEB) and clones were screened for chloramphenicol resistance.

*RNA band-shift assay and in vitro processing by RNase III*

Briefly, radioactive AsflhD was incubated with increasing concentrations of *flhD* mRNA under two conditions referred to as “native” (incubation in TMN buffer (20 mM Tris acetate, pH 7.5, 10 mM magnesium acetate, 100 mM sodium acetate for 5 min at 37◦C) and “full RNA duplex” (initial denaturation at 90°C for 2 min, then incubation in 1x TE buffer at 37°C for 30 min). The complexes were loaded on native polyacrylamide gels to control for hybridization efficiency or subjected to *in vitro* processing by RNase III of *E. coli*. RNase III digestion of free 5′-radiolabeled AsflhD, *flhD* or complexed AsflhD with *flhD* was performed at 37°C in TMN buffer containing 1 µg tRNA for 15 min with RNase III (Epicentre). Samples were loaded on denaturing polyacrylamide gels together with an RNA alkaline ladder as in (2).

*In vitro transcription assay*

Transcription assays were performed as described previously (3) in a buffer containing 20 mM Tris pH 8.0, 10 mM MgCl_2_, 100 mM KCl, 5 mM DTT and 200 µg/ml BSA. Open complex formation was carried out with 20 nM template, 100 nM holo-RNAP (core enzyme preincubated with a 4-fold excess of σ^70^ at 37°C for 20 min) and, where indicated, 100 nM CAP and 0.2 mM cAMP for 15 min at 37°C. Transcription was initiated by the addition of an RNTP mix (ATP, GTP, CTP at 200 µM, UTP at 20 µM and tracer amount of [α^32^P] UTP, about 1 µCie/lane) containing 150 µg/ml heparin and, where indicated, AsflhD or *flhD* transcripts. The AsflhD (256 nts) and *flhD* (308 nts) transcripts were the same as used for the *in vitro* RNase III processing assay. Transcription was stopped after 10 min at 37°C by the addition of 10 µl formamide with 10 mM EDTA, bromophenol blue and xylene cyanol. Samples were heated to 90°C for 5 min and separated on a 6% denaturing polyacrylamide gels (19/1). The purified RNAP core, σ^70^ and CAP proteins were the kind gifts of Annie Kolb. The size of the probed RNAs was estimated by comparison with migration of radio-labeled *Msp*1 digested pBR322 (NEB).

*Image treatment, quantifications and statistical analysis*

Statistical analysis for northern blot, β-galactosidase assays, *in vitro* transcription assays and swimming motility assays were performed as follows: for each tested condition, the mean of at least three independent biological replicates is indicated. Error bars represent the positive and negative calculated standard deviation. A significative difference was estimated by calculation of the p-value through ANOVA. P-values are indicated as follows; n.s. for p-values ≥0.05, * for p-values ≤0.05, ** for p-values ≤0.01, *** for p-values ≤0.001 and **** for p-values ≤0.0001. Blots were scanned using a Typhoon FLA 9500 scanner (GE Healthcare). Northern blots were analyzed using the ImageQuantTL v8.1 software (GE Healthcare). The acquired images were uniformly adjusted for their contrast before being cropped and assembled using the Image J software (<https://imagej.nih.gov/ij/>). It should be noted that blots with a separation line are assembled from one gel with intervening lanes omitted (*e.g.,* Fig. 1-C left) but are always from a unique membrane exposed with identical contrast parameters. Bands of interest were quantified and the abundance of the studied transcripts was normalized by comparison to the abundance of the M1 RNA (or 5S rRNA) for northern blot experiments. Of note, 5S was used in older experiments and replaced by M1 in our most recent experiments for technical reasons. Northern blots are representative of at least two biological replicates and quantified northern blot are the mean of at least three biological replicates. For the stability assays, normalized abundance of the studied mRNAs at the indicated times after rifampicin treatments were plotted using linear regression with a 90% confidence level, half-life and standard deviations were calculated using the Microsoft Excel software.

*Transcriptomic dataset comparison*

We compared the coordinates of asRNAs identified in the transcriptome of an *rnc* mutant (available in the ArrayExpress database at EMBL-EBI under accession number [E-MTAB-9507](https://www.ebi.ac.uk/arrayexpress/experiments/E-MTAB-9507/))(4) with previously published datasets as shown in Table 1. Antisense reads detected with the indicated coordinates in previous datasets are indicated (N; not detected, Y; detected). Of note, identical TSSs identified in the dataset of (5) for Ascrp, AsompR and AsflhD were detected in LB and in M63 during the exponential phase of growth and in LB during the stationary phase of growth. Data from previous studies were obtained from the supplemental table S1 for (6); supplemental table S5 for (7); in the dataset S01 for (8); in the dataset S03 for (5); in table S1 for (9). Genomic coordinates are indicated relative to the E. coli MG1655 reference genome U00096.3 (revised in 2016) or U00096.2 (for ease of comparison with datasets published before 2016 and aligned to U00096.2).

*Uncropped northern blots*

Northern blot membranes presented in Figure 8-ABC were loaded to compare each of the AsflhD mutant in a wt and in an *rnc* context. However, in this manuscript we do not present the effect of RNase III on the different AsflhD mutants in the expression of the motility cascade (*fliA*, *flgB* and *fliC* mRNAs) as we cannot distinguish direct and indirect effects of RNase III in the motility cascade. Similarly, the membrane presented in Figure S3-A was cropped to exclude the samples obtained in the *rnc* mutant. Uncropped northern blots membranes shown in Fig. 8-ABC and S3-A are shown below.


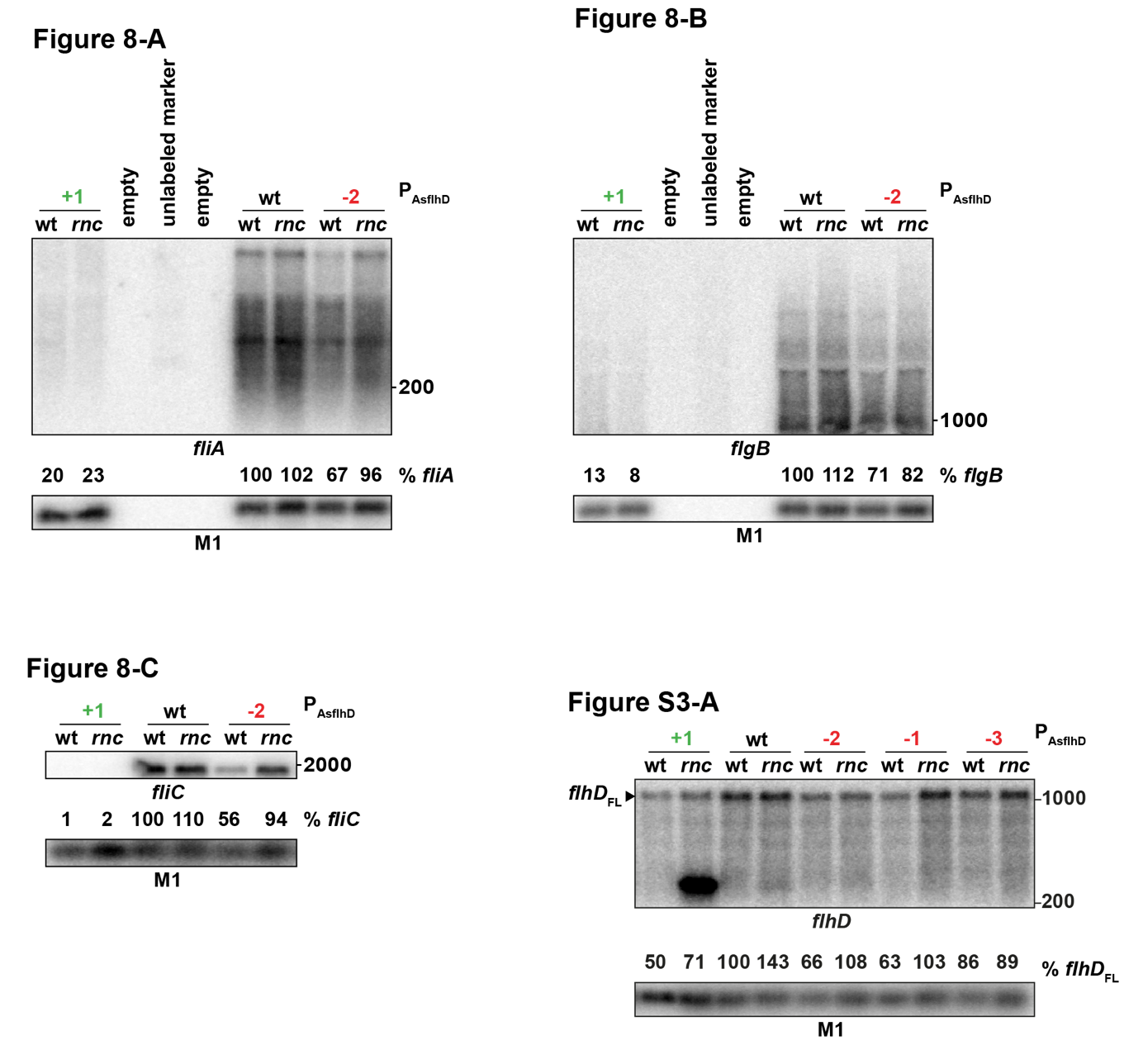


**Bibliography**

1. Jagodnik J, Chiaruttini C, Guillier M. 2017. Stem-Loop Structures within mRNA Coding Sequences Activate Translation Initiation and Mediate Control by Small Regulatory RNAs. Mol Cell 68:158-170.

2. Folichon M, Arluison V, Pellegrini O, Huntzinger E, Regnier P, Hajnsdorf E. 2003. The poly(A) binding protein Hfq protects RNA from RNase E and exoribonucleolytic degradation. Nucleic Acids Res 31:7302-7310.

3. Pennetier C, Oberto J, Plumbridge J. 2010. An antisense transcript from within the *ptsG* promoter region in *Escherichia coli*. J Mol Microbiol Biotechnol 18:230-240.

4. Maes A, Gracia C, Innocenti N, Zhang K, Aurell E, Hajnsdorf E. 2017. Landscape of RNA polyadenylation in *E. coli*. Nucleic Acids Res 45:2746-2756.

5. Thomason MK, Bischler T, Eisenbart SK, Forstner KU, Zhang A, Herbig A, Nieselt K, Sharma CM, Storz G. 2015. Global transcriptional start site mapping using differential RNA sequencing reveals novel antisense RNAs in *Escherichia coli*. J Bacteriol 197:18-28.

6. Dornenburg JE, DeVita AM, Palumbo MJ, Wade JT. 2010. Widespread Antisense Transcription in *Escherichia coli*. mBio 1.

7. Conway T, Creecy JP, Maddox SM, Grissom JE, Conkle TL, Shadid TM, Teramoto J, San Miguel P, Shimada T, Ishihama A, Mori H, Wanner BL. 2014. Unprecedented High-Resolution View of Bacterial Operon Architecture Revealed by RNA Sequencing. mBio 5.

8. Lybecker M, Zimmermann B, Bilusic I, Tukhtubaeva N, Schroeder R. 2014. The double-stranded transcriptome of *Escherichia coli*. Proc Natl Acad Sci U S A 111:3134-3139.

9. Ettwiller L, Buswell J, Yigit E, Schildkraut I. 2016. A novel enrichment strategy reveals unprecedented number of novel transcription start sites at single base resolution in a model prokaryote and the gut microbiome. BMC Genomics 17.
