# Supplementary material for: Regulatory Interplay between RNase III and Antisense RNAs in E. coli: the Case of AsflhD and FlhD, Component of the Master Regulator of Motility
Source: mBio. 2022 Aug 24;13(5):e00981-22. doi: 10.1128/mbio.00981-22 (PMC9600491; doi:10.1128/mbio.00981-22)
Supplement: TABLE S1 [file mbio.00981-22-s0010.docx]

***Table S1:*** ***strains, plasmids and primers used in this study***

| **Strains** | **Relevant genotype** | **Source** | |  |
| --- | --- | --- | --- | --- |
| N3433 | HfrH, *lacZ4, relA1, spoT1, thi1* | D. Apirion | |  |
| IBPC633 | N3433 *rnc105 nadB51::Tn10* | (1) | |  |
| C-5684 | *C-1a, rnc38::kanR* | (2) | |  |
| EM1321 | MG1655 Δ*lac*X174, *rnc14*::Tn10 | (3) | |  |
| KMT2444 | *rseA::kanR* | K. Thompson | |  |
| MG1655-B | Stable and highly motile isolate of MG1655 | This study | |  |
| ML65 | MG1655-B *rnc38::kanR* | This study | |  |
| N3433-pnp | MG1655-B *rnc14::Tn10* | (4) | |  |
| IBPC633-pnp | N3433 *pnp::kanR, rnc105 nadB51::Tn10* | (4) | |  |
| N3431 | N3433 *rne3071^ts^* | (5) | |  |
| IBPC637 | N3433 *rne3071^ts^, rnc105 nadB51::Tn10* | (1) | |  |
| N3433-fliC::lacZ-trans | P*_fliC_*-*lacZ cmR* | (6) | |  |
| JW1881 | BW25113 *flhD::kanR* | (7) | |  |
| MG2114 | MG1655 mini *λ, tetR,* *rrnB*T2-*cat*-*sacB-lacZ_(-17+1)_* | M. Guillier | |  |
| ML69 | MG1655-B, *flhD*::*cat-sacB* | This study | |  |
| ML611 | ML69 *rnc38::kanR* | This study | |  |
| ML71 | ML69 mini λ, *tetR* | This study | |  |
| MG2118 | MG2114 P_AsphoP(wt)-150+15_-*lacZ*_-17_ | This study | |  |
| MG2120 | MG2114 P_AsphoP(-4)-150+15_-*lacZ*_-17_ | This study | |  |
| ML73 | MG1655-B P_AsflhD(-2)_ | This study | |  |
| ML609 | MG1655-B P_AsflhD(-1)_ | This study | |  |
| ML610 | MG1655-B P_AsflhD(-3)_ | This study | |  |
| ML241 | MG1655-B P_AsflhD(+1)_ | This study | |  |
| ML75 | ML73 *rnc38::kanR* | This study | |  |
| ML341 | ML241 *rnc38::kanR* | This study | |  |
| MG2114-P_AsflhD_ | MG2114 P_AsflhD(wt)-165+15_-*lacZ*_-17_ | This study | |  |
| ML239 | MG2114 P_AsflhD(-2)-165+15_-*lacZ*_-17_ | This study | |  |
| ML604 | MG2114 P_AsflhD(-1)-165+15_-*lacZ*_-17_ | This study | |  |
| ML605 | MG2114 P_AsflhD(-3)-165+15_-*lacZ*_-17_ | This study | |  |
| ML218 | MG2114 P_AsflhD(+1)-165+15_-*lacZ*_-17_ | This study | |  |
| ML279 | MG2114 P_AsflhD(wt)_ *rseA::kanR* | This study | |  |
| ML219 | MG2114 P*_flhD-108+300_*-*lacZ*_+52_ | This study | |  |
| ML221 | MG2114 P*_flhD-108+300_*-*lacZ*_+52,_ P_AsflhD(-2)_ | This study | |  |
| ML226 | MG2114 P*_flhD-108+300_*-*lacZ*_+52_, P_AsflhD(+1)_ | This study | |  |
| ML233 | MG2114 P*_tet_*-*flhD*_+1+300_-*lacZ*_+52_ | This study | |  |
| ML235 | MG2114 P*_tet_*-*flhD*_+1+300_-*lacZ*_+52_, P_AsflhD(-2)_ | This study | |  |
| ML237 | MG2114 P*_tet_*-*flhD*_+1+300_-*lacZ*_+52_, P_AsflhD(+1)_ | This study | |  |
|  | | |  |  |
| ML616 | MG1655-B, P*_fliC_*-*lacZ*_-_ | This study | |  |
| ML617 | ML73, MG1655-B P_AsflhD(-2)_, P*_fliC_*-*lacZ*_-_ | This study | |  |
| ML618 | ML609, MG1655-B P_AsflhD(+1)_, P*_fliC_*-*lacZ*_-_ | This study | |  |
| ML619 | ML610, MG1655-B P_AsflhD(-3)_, P*_fliC_*-*lacZ*_-_ | This study | |  |
| ML615 | ML241, MG1655-B P_AsflhD(+1)_, P*_fliC_*-*lacZ*_-_ | This study | |  |
| ML621 | ML616 *flhD::kanR* | This study | |  |

| **Plasmids** | **Relevant genotype** | **Source** |
| --- | --- | --- |
| pCA24N control | *lacI^q^, cmR* | (8) |
| pCA24N AsflhD | *P_tac_-AsflhD-rrnbT2, lacI^q^, cmR* | This study |

|  | |  |
| --- | --- | --- |
| **Primer** | **Sequence** | **Information** |
|  |  |  |
| **RNA-seq** | | |
| TSS | GAGAGGACCUAGGGAA |  |
| PSS | AGGGACCAGUAGGAAA |  |
|  |  |  |
| **Northern blot** | | |
| mcrp | AAACCGCAAACAGACCCGA | *crp* |
| T7crp | TAATACGACTCACTATAGGGCCCTCTTCAAACAGGC |  |
| mascrp | GGCCCTCTTCAAACAGGC | Ascrp |
| T7ascrp | TAATACGACTCACTATAGGGAAACCGCAAACAGACCCGA |  |
| masompR1 | GGAAAGATTCACGAGTCAGC | *ompR* |
| T7asOmpR2 | TAATACGACTCACTATAGGGAGTACAAACAATGCAAG |  |
| mompR3 | GGGAGTACAAACAATGCAAG | AsompR |
| T7ompR4 | TAATACGACTCACTATAGGGAAAGATTCACGAGTCAGC |  |
| mphoP1 | GGTTGTTGAAGACAATGCG | *phoP* |
| T7phoP2 | TAATACGACTCACTATAGGGCGCCATCACCTCTTCAA |  |
| masphoP2 | GCGCCATCACCTCTTCAA | AsphoP |
| T7asphoP3 | TAATACGACTCACTATAGGGATGCGCGTACTGGTTGTTGAA |  |
| mflhD | GCAGCTTATCGCAACTATTC | *flhD* 5' (5'-UTR) |
| T7flhD | TAATACGACTCACTATAGGGAGGTATGCATTATTCCC |  |
| mflhD14 | GCGTTTGATTGTTCAGGACA | *flhD* 3' (ORF) |
| T7flhD15 | TAATACGACTCACTATAGGGCCAGTTGATTGGTTTCTGCC |  |
| masflhD | GGAGGTATGCATTATTCCC | AsflhD |
| T7asflhD | TAATACGACTCACTATAGGGCAGCTTATCGCAACTATTC |  |
| mfliA | ACAAGGAACCGCATTATCAACT | *fiA* |
| T7fliA | TAATACGACTCACTATAGGGAGAAGAGCTGGCTGTTATTG |  |
| mfliC | TGGCACAAGTCATTAATACCAAC | *fliC* |
| T7fliC | TAATACGACTCACTATAGGGCGGTCAATTTCATCCAGAC |  |
| mflgB | TTTGATACCTGCGGAGGAGA | *flgB* |
| T7flgB | TAATACGACTCACTATAGGGTCATGCCTTTGATTTGCCCG |  |
| M1 | GCTCTCTGTTGCACTGGTCG | M1 |
| 5S | ACTACCATCGGCGCTACGGC | 5S |
|  |  |  |
| **cRT-PCR** | | |
| mflhD2 | GCAGCTTATCGCAACTATTC |  |
| mflhD6 | ATCACGGGGTGCGGTGAAA |  |
| masflhD10 | GCCCCGGTAAAAAATTAGC |  |
|  |  |  |
| **RNase III maturation** | | |
| Up-T7-flhD+308 | TAATACGACTCACTATAGGGATTTAGGAAAAATCTTAGATAAGTG |  |
| Down-flhD | CCGAGACGAAACATAGCGG |  |
| Up-T7-AsflhD | TAATACGACTCACTATAGGGTCAGCAACTCGGAGGTATGCAT |  |
| Down-AsflhD | GTTGTATGTGCGTGTAGTGAC |  |
|  |  |  |
| **Mutants** |  |  |
| 5'-cat-sacB | TTGTGTGATCTGCATCACGCATTATTGAAAATCGCAGCCCCAAAATGAGACGTTGATCGGCACG | instertion of *cat-sacB* in *flhD* |
| 3'-cat-sacB | GCGGACGCTTTGTCCTGAACAATCAAACGCTGTGCAAGTAGTATCAAAGGGAAAACTGTCCATAT |  |
| Up-cat-sacB | GTAATTGAGTGTTTTGTGTGATCTGCATCACGCATTATTGAAAATCGCAGCCCC | Endogenous AsflhD mutant |
| Down-PAsflhD-2 | ACATAGCGGACGCTTTGTCCTGAACAATCAAACGCTGTGCAAGcAaTAAATATGACAAGTTGATGTCGTAAATGTGT | P_AsflhD-2_ |
| Down-PAsflhD-1 | ACATAGCGGACGCTTTGTCCTGAACAATCAAACGCTGTGCAAGTAGTAAATATGACAAGTTGATGTCgTAAATGTGT | P_AsflhD-1_ |
| Down-PAsflhD-3 | ACATAGCGGACGCTTTGTCCTGAACAATCAAACGCTGTGCAAGcAaTAAATATGACAAGTTGATGTCgTAAATGTGT | P_AsflhD-3_ |
| Down-PAsflhD+ | ACATAGCGGACGCTTTGTCCTGAACAATCAAACGCTGTGCAAGTAGTAAATATGACAAGTTGATGTtATAAATGTGT | P_AsflhD_^+^ |
|  |  |  |
| **Reporter** | | |
| Up-PAsphoP | GGCGCAGAAGGCCATCCTGACGGATGGCCTTTTTGCGTTTATTAATAGATAATTCACGGCGAGAG | P_AsphoP-150+15_-*lacZ*_-17_ |
| Down-PAsphoP | CAGTGAATCCGTAATCATGGTCATAGCTGTTTCCTGTGTGAGTATTAAGTGCCGGTGCTGATG |  |
| Up-PAsphoP- | CTCGATATGAAACGGTTTAGTCACGTAGTCATCAGCACCGGCACTTAATA | P_AsphoP_^-^_-150+15_-*lacZ*_-17_ |
| Down-PAsphoP- | CTACGTGACTAAACCGTTTCATATCGAGGAGGTGATGGCGCGAATGCAG |  |
| Up-PAsflhD-165 | GGCGCAGAAGGCCATCCTGACGGATGGCCTTTTTGCGTTTACCAGTTGATTGGTTTCTGC | P_AsflhD-165+15_-*lacZ*_-17_ |
| Down-PAsflhD+15 | CAGTGAATCCGTAATCATGGTCATAGCTGTTTCCTGTGTGACCTCCGAGTTGCTGAAACAC |  |
| Up-PflhD-108 | GGCGCAGAAGGCCATCCTGACGGATGGCCTTTTTGCGTTTGATCTGCATCACGCATTATTG | P*_flhD_*_-108+300_-*lacZ*_+28_ |
| Down-PflhD+300 | TAACGCCAGGGTTTTCCCAGTCACGACGTTGTAAAACGACAAACATAGCGGACGCTTTG |  |
| Up-Ptet-flhD+1 | AGAGATTGACATCCCTATCAGTGATAGAGATACTGAGCACGATTTAGGAAAAATCTTAGA | P*_tet_*-*flhD*_+1+300_-*lacZ*_+28_ |
|  |  |  |
| **Plasmid** |  |  |
| pCA24N AsflhD+1 | GTGAGCGGATAACAATTATAATAGATTCTCAGCAACTCGGAGGTATGC | AsflhD_+1+220_ |
| AsflhD +220-rrnBT2 | CATGGATCCGACGGCCAGTAAACGCAAAAAGGCCATCCGTCAGGATGGCCTTCTGATTTAGGAAAAATCTTAGA |  |
| pCA24N-XhoI-AsflhD+1 | CGTCTTCACCTCGAGAAATCATAAAAAATTTATTTGCTTTGTGAGCGGATAACAATTATA | Xho1-AsflhD_+1+220_-HindIII |
| pCA24N-HindIII-AsflhD+220 | GTCCAAGCTCAGCTAATTAAGCTTCATGGATCCGACGGCCAGTA |  |
|  |  |  |
| **In vitro transcription** |  |  |
| Up-flhD-ivt | AGAGATTGACATCCCTATCAGTGATAGAGATACTGAGCACGATTTAGGAAAAATCTTAGA |  |
| Up-Ptet-ivt | GGCGCAGAAGGCCATCCTGACGGATGGCCTTTTTGCGTTTGATCTGCATCACGCATTATTG |  |
| Down-flhD-ivt | ACCAGTTGATTGGTTTCTGCC |  |

**Bibliography**

1. Regnier, P. and Hajnsdorf, E. (1991) Decay of mRNA encoding ribosomal protein S15 of Escherichia coli is initiated by an RNase E-dependent endonucleolytic cleavage that removes the 3' stabilizing stem and loop structure. *J Mol Biol*, **217**, 283-292.

2. Carzaniga, T., Briani, F., Zangrossi, S., Merlino, G., Marchi, P. and Dehò, G. (2009) Autogenous regulation of *Escherichia coli* polynucleotide phosphorylase expression revisited. *J Bacteriol*, **191**, 1738-1748.

3. Massé, E., Escorcia, F.E. and Gottesman, S. (2003) Coupled degradation of a small regulatory RNA and its mRNA targets in *Escherichia coli*. *Gene & Dev.*, **17**, 2374-2383.

4. Fontaine, F., Gasiorowski, E., Gracia, C., Ballouche, M., Caillet, J., Marchais, A. and Hajnsdorf, E. (2016) The small RNA SraG participates in PNPase homeostasis. *RNA*, **22**, 1560-1573.

5. Goldblum, K. and Apirion, D. (1981) Inactivation of the ribonucleic acid-processing enzyme ribonuclease E blocks cell division. *J Bacteriol*, **146**, 128-132.

6. Maes, A., Gracia, C., Brechemier, D., Hamman, P., Chatre, E., Lemelle, L., Bertin, P.N. and Hajnsdorf, E. (2013) Role of polyadenylation in regulation of the flagella cascade and motility in *Escherichia coli*. *Biochimie*, **95**, 410-418.

7. Baba, T., Ara, T., Hasegawa, M., Takai, Y., Okumura, Y., Baba, M., Datsenko, K.A., Tomita, M., Wanner, B.L. and Mori, H. (2006) Construction of *Escherichia coli* K‐12 in‐frame, single‐gene knockout mutants: the Keio collection. *Mol. Systems Biol.*, **2**.

8. Kitagawa, M., Ara, T., Arifuzzaman, M., Ioka-Nakamichi, T., Inamoto, E., Toyonaga, H. and Mori, H. (2005) Complete set of ORF clones of *Escherichia coli* ASKA library (A Complete Set of *E. coli* K-12 ORF Archive): Unique Resources for Biological Research. *DNA Res.*, **12**, 291-299.
